# Neuron Morphology Influences Axon Initial Segment Plasticity^1^,^2^,^3^

**DOI:** 10.1523/ENEURO.0085-15.2016

**Published:** 2016-02-13

**Authors:** Allan T. Gulledge, Jaime J. Bravo

**Affiliations:** 1 Department of Physiology and Neurobiology, Geisel School of Medicine at Dartmouth, Dartmouth-Hitchcock Medical Center, Lebanon, New Hampshire 03756; 2 Thayer School of Engineering at Dartmouth, Hanover, New Hampshire 03755

**Keywords:** action potential, AIS plasticity, axon, axon initial segment, dendrite, sodium channel

## Abstract

In most vertebrate neurons, action potentials are initiated in the axon initial segment (AIS), a specialized region of the axon containing a high density of voltage-gated sodium and potassium channels. It has recently been proposed that neurons use plasticity of AIS length and/or location to regulate their intrinsic excitability. Here we quantify the impact of neuron morphology on AIS plasticity using computational models of simplified and realistic somatodendritic morphologies. In small neurons (e.g., dentate granule neurons), excitability was highest when the AIS was of intermediate length and located adjacent to the soma. Conversely, neurons having larger dendritic trees (e.g., pyramidal neurons) were most excitable when the AIS was longer and/or located away from the soma. For any given somatodendritic morphology, increasing dendritic membrane capacitance and/or conductance favored a longer and more distally located AIS. Overall, changes to AIS length, with corresponding changes in total sodium conductance, were far more effective in regulating neuron excitability than were changes in AIS location, while dendritic capacitance had a larger impact on AIS performance than did dendritic conductance. The somatodendritic influence on AIS performance reflects modest soma-to-AIS voltage attenuation combined with neuron size-dependent changes in AIS input resistance, effective membrane time constant, and isolation from somatodendritic capacitance. We conclude that the impact of AIS plasticity on neuron excitability will depend largely on somatodendritic morphology, and that, in some neurons, a shorter or more distally located AIS may promote, rather than limit, action potential generation.

## Abstract (precis)

In most vertebrate neurons, action potentials are initiated in the axon initial segment (AIS), a specialized region of the axon containing a high density of voltage-gated sodium and potassium channels.

## Visual Abstract

**Figure:**
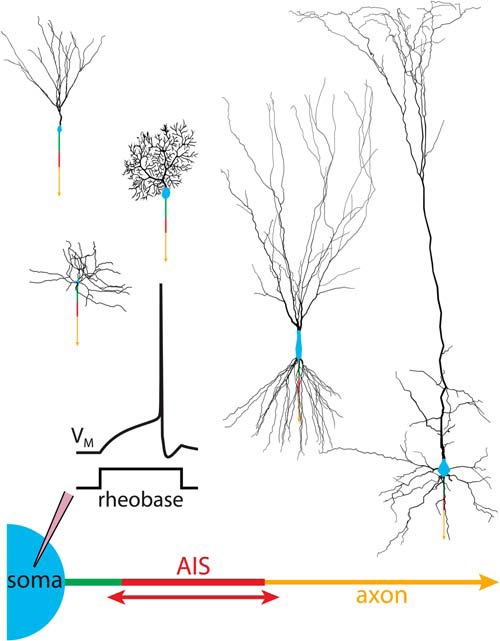

## Significance Statement

Action potentials in vertebrate neurons are initiated in the axon initial segment (AIS), a specialized region of axon containing a high density of voltage-gated sodium channels. It has been proposed that neurons regulate their intrinsic excitability via plastic changes in AIS length and/or location. Here we use computational modeling to quantify the impact of AIS plasticity across a range of simplified and realistic neuron morphologies. We demonstrate that somatodendritic morphology will dictate both the magnitude and direction of excitability changes occurring in response to AIS plasticity. Excitability of large neurons is enhanced when the AIS is longer and/or distal from the soma, while small neurons are most excitable when the AIS is of intermediate length and/or adjacent to the soma.

## Introduction

Action potentials in most vertebrate neurons are initiated in a specialized region of proximal axon known as the axon initial segment (AIS). To promote action potential initiation, the AIS is enriched with voltage-gated sodium and potassium channels that occur at densities approximately 50 times greater than those at the soma (Kole et al., 2008). Previous studies have revealed cell type-dependent heterogeneity in the architecture of the AIS, including diversity in AIS length and location relative to the soma. For instance, in the nucleus laminaris of the avian auditory system, neurons processing high-frequency sounds typically have a short (∼10 µm) AIS located distally (∼45 µm) from the soma, while neurons responsive to lower-frequency sounds typically have a longer (∼25 µm) and more proximal (within ∼10 µm of the soma) AIS (Kuba et al., 2006).

It has recently been proposed that dynamic regulation of AIS architecture provides an intrinsic homeostatic mechanism to regulate neuron excitability in response to chronic changes in synaptic drive (Grubb et al., 2011). In cultured hippocampal neurons, for instance, prolonged periods of enhanced excitatory drive reversibly translocate the AIS away from the soma (Grubb and Burrone, 2010; Evans et al., 2013; Muir and Kittler, 2014). These shifts in AIS location were correlated with decreased intrinsic excitability, as reflected in higher somatic current thresholds (rheobase currents) for action potential initiation (Grubb and Burrone, 2010; Evans et al., 2013). Similarly, in both the auditory (Kuba et al., 2014) and visual (Gutzmann et al., 2014) systems, postnatal sensory experience sculpts AIS morphology, while experimental manipulations of sensory input drive dynamic modifications of AIS architecture (Gutzmann et al., 2014; Kuba et al., 2014) that correlate with changes in neuron excitability (Kuba et al., 2010).

However, the impact of AIS morphology on intrinsic excitability is not well understood. In general, a longer AIS, containing more sodium channels, is expected to enhance excitability (Kuba et al., 2010), while translocation of the AIS to distal locations experiencing greater voltage attenuation of synaptic signals is predicted to reduce excitability (Grubb and Burrone, 2010). Yet the AIS does not exist in isolation; somata and dendrites generate capacitive and conductive loads that influence excitability by facilitating the escape of inward sodium current from the AIS (Eyal et al., 2014). Therefore, the most excitable AIS length or location will depend on the balance of its electrical accessibility to depolarizing drive and electrical isolation from somatodendritic conductance loads (Kuba et al., 2006; Baranauskas et al., 2013).

The diversity of dendritic and axonal morphologies complicates predictions of how changes to AIS architecture will impact neuron excitability. To better understand the interaction between somatodendritic morphology and AIS plasticity, we paired models of simplified and realistic (reconstructed) neurons with a variety of AIS architectures. Our results demonstrate that AIS performance depends heavily on somatodendritic morphology, and suggest that, in some neurons, plastic changes in AIS length and location that are typically predicted to reduce neuron excitability may instead promote action potential initiation.

## Materials and Methods

Simulations were performed using NEURON 7.3 software (Carnevale and Hines, 2006). Neuronal morphologies included simple “ball-and-stick” models consisting of a cylindrical soma (20 µm in length × 20 µm in diameter) attached to a variable number of tapering dendrites (300 µm long, tapering from 2.5 to 0.5 µm in diameter), a medium spiny neuron (Martone et al., 2002), a hippocampal dentate granule cell (Schmidt-Hieber et al., 2007), a cerebellar Purkinje cell (Roth and Häusser, 2001), a layer 5 (L5) pyramidal neuron from the somatosensory cortex (Stuart and Spruston, 1998), and a hippocampal CA3 pyramidal neuron (Hemond et al., 2009). Unless otherwise noted, cytoplasmic resistivity (R_i_), specific membrane capacitance (C_M_), and specific membrane resistivity (R_M_) were set to 100 Ω · cm, 1 µF/cm^2^, and 15,000 Ω · cm^2^, respectively, with the reversal potential for the passive conductance set to −70 mV (Table 1). Realistic morphologies were attached to myelinated (Purkinje Cell and L5 pyramidal neuron) or unmyelinated (medium spiny neuron, dentate granule cell, and CA3 pyramidal neuron) axons (see below), as appropriate. In some simulations, the electrical impact of dendritic spines in realistic neuron morphologies was simulated by doubling dendritic C_M_ and halving dendritic R_M_ (Holmes, 1989). In simulations for movies involving the L5 neuron, apical dendrites included voltage-activated sodium and potassium conductances, and a hyperpolarization- and cyclic nucleotide-gated cation conductance (Hallermann et al., 2012) whose conductance density increased exponentially according to the following function: *y*_0_ + *A* exp (*d*/λ), where *y*_0_ = −2 pS/μm^2^, *A* = 4.29 pS/μm^2^, λ = 324 μm, and *d* = distance from soma in micrometers (Kole et al., 2006). These conductances were left out of the L5 neuron in most simulations to standardize dendritic membrane properties across all realistic neuron morphologies. The presence (e.g., in the ball-and-stick neurons and layer 5 pyramidal neuron for movies) or absence (e.g., in the reconstructed neuron morphologies) of active conductances in the dendrites did not qualitatively alter the impact of AIS plasticity.

**Table 1:** Model parameters

Compartment	Dimensions (length × diameter)	Segments (*n*)	Max g_Na^+^_/g_K^+^_ (pS/µm^2^)
Dendrites	300 µm, tapered from 2.5 to 0.5 µm	101	Na^+^, linear decrease, 100-20; K^+^, linear decrease, 100-20
Somata	20 × 20 µm	11	Na^+^, 100; K^+^, 100
Proximal axon	0-100 × 1.5 µm	∼1/µm	Na^+^, 100; K^+^, 100
AIS	5-100 × 1.5 µm	∼1/µm	Na^+^, 8000; K^+^, 2000
Myelin segments (20)	100 × 1 µm	21	Passive; C_M_/10, R_M_ · 10
Nodes of Ranvier (20)	1 × 1.5 µm	3	Na^+^, 2,667; K^+^, 667
Unmyelinated axon	2000 × 1 µm	401	Na^+^, 300; K^+^, 60
Axon endpoint	10 × 10 µm	11	Passive; C_M_ · 2, R_M_/2
MSN, DGC, Purkinje, L5, and CA3 neurons	Reconstructed morphologies	∼1/µm	Somata: Na^+^, 100; K^+^, 100; Dendrites, passive

DGC, Dentate granule cell; E_pas_, reversal potential for passive leak conductance; MSN, medium spiny neuron. General parameters: R_M_ = 15 kΩ · cm^2^; C_M_ = 1 µF/cm^2^; R_i_ = 100 Ω · cm; E_pas_ = −70 mV; time steps, 0.1 or 1 µs; nominal temperature, 37°C.

**Figure 1.**
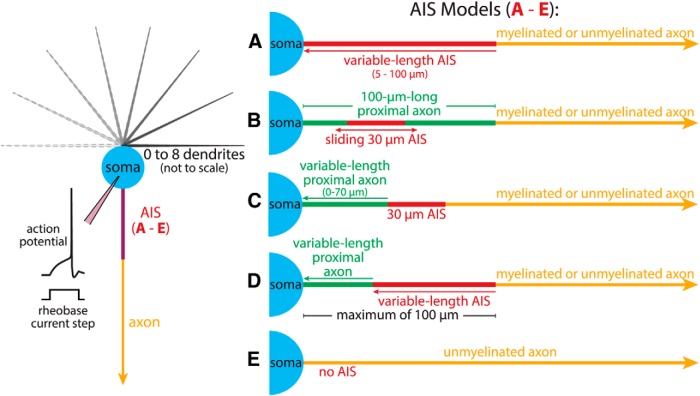
Diagrams of somatodendritic (left) and axonal (right) compartments in a model neuron. Somata (20 × 20 µm) were attached to a variable number of dendrites (300 µm long, tapering from 2.5 to 0.5 µm in diameter), and to one of five AISs. The AIS in Model A was variable in length (5-100 µm), while AIS Model B had a 30-µm-long AIS that was translocatable (from 0 to 70 µm from the soma) within a 100-µm-long proximal axon segment (1.5 µm diameter) having somatic membrane properties. AIS Model C had a similar 30 µm AIS fixed to the axon proper on one end, and to a variable amount of proximal axon (0-70 µm long, 1.5 µm diameter, with somatic membrane properties) bridging itself and the soma. AIS Model D was a hybrid model combining a variable-length proximal axon with a variable-length AIS (maximum combined length was 100 µm). Finally, AIS Model E lacked an AIS altogether and consisted of an unmyelinated axon attached directly to the soma. AIS Models A through D were attached to myelinated or unmyelinated axons (see Materials and Methods). Inset at left illustrates the simulation setup in which rheobase current injections at the soma (40 ms duration) initiate action potentials.

The model neurons described above were attached to one of five AIS structures (AIS Models A, B, C, D, or E; Fig. 1). AIS Model A consisted of a variable-length AIS (5-100 µm, 1.5 µm diameter) containing either uniform maximum conductance densities for sodium (8000 pS/µm^2^, unless otherwise noted) and potassium (2000 pS/µm^2^; Hallermann et al., 2012), or adjusted conductance densities set to conserve, across all AIS lengths, the equivalent maximal total sodium and potassium conductances as exist in a 30-µm-long AIS having the sodium and potassium conductance densities stated above. Unless otherwise noted, voltage-gated sodium and potassium conductances were NEURON implementations of currents originally published by Mainen and Sejnowski (1996; available from entry 2488 at ModelDB at http://modeldb.yale.edu/). The variable-length AIS bridged the soma with a myelinated or unmyelinated axon (see below). AIS Model B consisted of a fixed 100-µm-long proximal axon (1.5 µm diameter) having somatic membrane properties that connected the soma to either a myelinated or unmyelinated axon. Within this proximal axon was a 30-µm-long AIS (maximum sodium and potassium conductance densities typically set to 8000 and 2000 pS/µm^2^, respectively) that could be positioned at various distances from the soma (i.e., from 0 to 70 µm from the soma). An advantage of AIS Model B was that gross morphologies, total neuron surface areas, and total membrane conductances were conserved and independent of AIS location. A morphologically more realistic model, AIS Model C, consisted of a 30 µm AIS attached to a myelinated or unmyelinated axon on one end, and to a variable amount of soma-like proximal axon (0-70 µm, 1.5 µm diameter) between itself and the soma. AIS Model D was a hybrid of AIS Models A and C, having a variable-length AIS (5-70 µm) paired with a variable-length proximal axon (0-95 µm), with the combined structure limited to a maximum of 100 µm. Finally, AIS Model E, which lacked an AIS altogether, consisted of an unmyelinated axon attached directly to the soma.

In some simulations the ratio of AIS to somatic sodium conductances (normally 80:1) was lowered. This was accomplished primarily by proportionally lowering the maximal AIS sodium and potassium conductance densities toward somatic values (normally 100 pS/µm^2^ for each; Table 1). To preserve action potential initiation in the AIS at the 5:1 AIS-to-soma conductance ratio, maximal somatic sodium and potassium densities were raised to 200 pS/µm^2^ (each), and maximal AIS densities of sodium and potassium conductances were set to 1,000 and 250 pS/µm^2^, respectively. In simulations in which AIS-to-soma ratios were modified while the 30 µm AIS was moved distally from the soma, no active conductances were inserted into the proximal axon bridging the AIS to the soma.

Myelinated axons consisted of twenty 100-µm-long passive segments having low membrane capacitance (C_M_ = 0.1 µF/cm^2^) and high resistivity (R_M_ = 150 kΩ · cm^2^), interspersed with 20 nodes of Ranvier (1-µm-long, diameter of 1.5 µm) having somatic passive properties and maximum sodium and potassium conductance densities set to one-third of those in the AIS (i.e., 2667 and 667 pS/µm^2^, respectively; Table 1). Unmyelinated axons were 2000 µm long (1 µm diameter) and contained a uniform distribution of sodium (300 pS/µm^2^) and potassium (60 pS/µm^2^) conductances. All axons terminated with a 10 × 10 µm passive endpoint having a C_M_ of 2 µF/cm^2^ and an R_M_ of 7500 Ω · cm^2^, although control simulations found that the presence or absence of axon endpoints had no effect on rheobase currents at the soma (data not shown).

To compare the effective isolation of AIS compartments from somatodendritic capacitance, we computed a “C_M_ isolation index.” To do this, for each AIS location we measured the percentage change in rheobase current produced by adding 5% to somatodendritic capacitance (i.e., after setting C_M_ in those compartments to 1.05 µF/cm^2^). Using the following formula, we calculated the C_M_ isolation index, which reflects, for any given AIS location, the percentage by which the impact of changes to somatodendritic capacitance on rheobase current (as detected to the nearest 1 fA) are reduced relative to the maximal impact on rheobase current that occurs when the AIS is adjacent to the soma (where the C_M_ isolation index is 0), as follows:

$${\text{C}}_{\text{M}}\;\text{isolation index}=\frac{\%\;\text{change in rheobase when AIS is at}\;0\;\mu \text{m}-\;\text{local \% change in rheobase}}{\%\;\text{change in rheobase when AIS is at}\;0\;\mu \text{m}}\times \;100$$

Unless otherwise noted, simulations were performed with 1 µs time steps, and the rheobase determined to the nearest 100 fA in response to somatic current injection from action potentials occurring in the axon, regardless of whether these AIS action potentials successfully propagated to the soma. Indeed, it is important to note that we tested a wide range of AIS lengths and locations to fully explore the impact of somatodendritic geometry and AIS architecture on neuron excitability, and that in some models in which the AIS was remote from the soma (up to 95 µm away), action potentials failed to propagate into the soma. For any given somatodendritic morphology, when more than one AIS morphology had equal minimal thresholds (at 100 fA resolution), the shortest or most proximal AIS having that minimal threshold was recorded as having the lowest rheobase. Simulations assumed a nominal temperature of 37°C, unless otherwise noted. Averaged data are presented as the mean ± standard deviation.

## Results

### Somatodendritic morphology influences AIS performance

**Figure 2.**
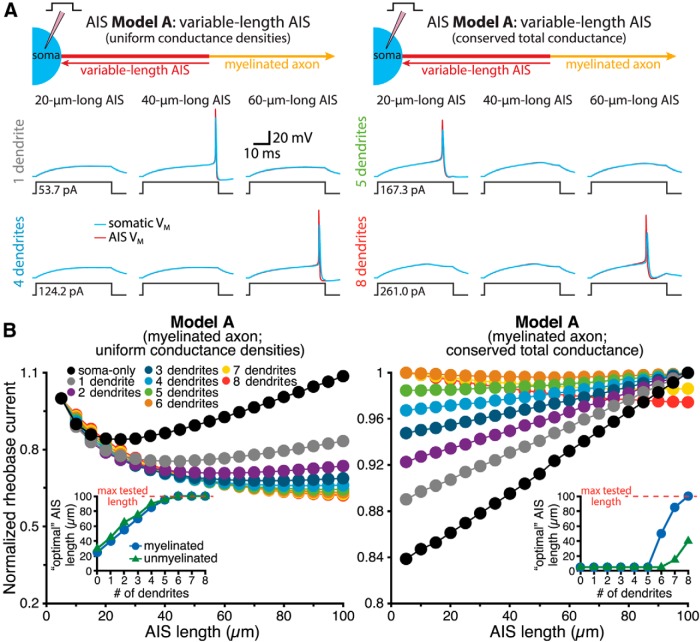
Interaction of neuron size and AIS length in regulating action potential threshold. ***A***, Diagrams (top) of ball-and-stick neurons having a variable-length AIS (AIS Model A) with uniform maximum sodium and potassium conductance densities (left), or with conserved (i.e., length-independent) total conductances (right). Voltage traces (below) show somatic (blue) and AIS (red) responses to somatic current injections in neurons with the indicated number of dendrites and AIS lengths of 20 (left), 40 (middle), or 60 µm (right). ***B***, Plots of normalized rheobase currents in neurons having the indicated numbers of dendrites and AIS lengths, with uniform conductance densities (left) or conserved total conductances (right). Inset, Plots of optimal (i.e., lowest rheobase current) AIS length vs the number of dendrites in model neurons having myelinated or unmyelinated axons, as indicated. Larger neurons had longer optimal AIS lengths. To facilitate comparisons across neurons, rheobase currents from the soma-only neuron having uniform AIS conductance densities were normalized to the rheobase occurring when the AIS was adjacent to the soma, as at very long AIS lengths, rheobase increased above this level in the soma-only model.

We first tested the interaction of somatodendritic morphology and AIS length in regulating rheobase current thresholds in simplified ball-and-stick neurons having zero to eight dendrites and a variable-length (5-100 µm) AIS attached to a myelinated or unmyelinated axon (Fig. 1; AIS Model A). For each morphological configuration, rheobase currents were determined to the nearest 100 fA using somatic current injections (40 ms duration). In some models, the AIS maintained constant maximal sodium (8000 pS/µm^2^) and potassium (2000 pS/µm^2^) conductance densities (Fig. 2*A*, left), while in other models maximum conductance densities were adjusted by AIS length, such that total conductances were length independent, and equivalent to those in a 30-µm-long AIS having sodium and potassium conductances set to 8000 and 2000 pS/µm^2^, respectively (Fig. 2*A*, right). Given that high sodium channel densities in the AIS promote action potential initiation (Kole et al., 2008), we predicted that a longer AIS, with greater total sodium conductance, would be associated with a lower rheobase threshold. Surprisingly, this was not always the case. In smaller neurons (i.e., those with few or no dendrites), lowest rheobase current thresholds occurred when the AIS was of intermediate lengths (Fig. 2, left panels). “Optimal” AIS length (i.e., the length at which rheobase currents were minimal) was directly related to somatodendritic size: the larger the neuron, the longer the optimal AIS length. This was true in both myelinated and unmyelinated neurons (Fig. 2*B*, left inset).

Similarly, when total maximum sodium conductance in the AIS was preserved (i.e., was length independent), optimal AIS length was again dependent on somatodendritic size (Fig. 2, right panels). Rheobase currents were lowest in small- and medium-sized neurons when the AIS was short (5 µm long), with correspondingly high maximal conductance densities (e.g., 48,000 and 12,000 pS/µm^2^ for sodium and potassium conductances, respectively, for the 5 µm AIS). In these neurons, rheobase currents increased as the AIS was lengthened and conductance densities diminished. However, in larger neurons (i.e., those having more than five dendrites), rheobase currents continued to decrease as the AIS elongated with progressively lower sodium and potassium conductance densities (Fig. 2*B*, right inset). These results demonstrate that somatodendritic architecture independently influences AIS performance, with action potential generation in smaller neurons facilitated when the AIS is short and contains a high density of sodium channels, while excitability in larger neurons is promoted when the AIS is longer, even at the expense of high conductance densities.

**Figure 3.**
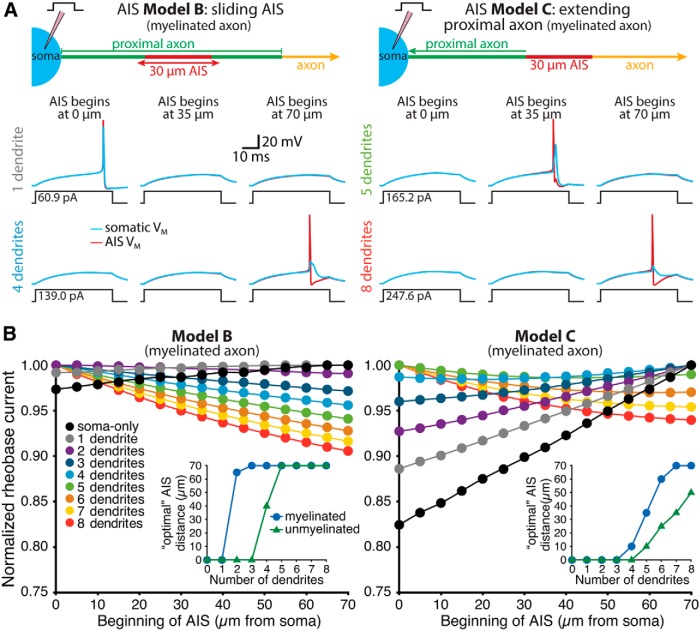
Dendritic architecture influences the impact of AIS location on excitability. ***A***, Diagrams (top) of the AIS of ball-and-stick models having a translocating AIS within the proximal axon (AIS Model B, left) or an AIS connected to the axon proper and positioned at variable distance from the soma (AIS Model C, right). Voltage traces show somatic (blue) and AIS (red) responses to somatic current injections in neurons in which the AIS was positioned 0 µm from the soma (left), 35 µm from the soma (middle), or 70 µm from the soma (right). Data are from models with one or four dendrites (AIS Model B), or five or eight dendrites (AIS Model C), as indicated. Note that in both model configurations the addition of dendrites increased the rheobase current, but shifted the most excitable AIS locations distally. ***B***, Plots of normalized rheobase currents in myelinated neurons having the indicated number of dendrites and AIS locations for neurons with AIS Model B (left) or AIS Model C (right). Inset, Plots of optimal (i.e., lowest rheobase current) AIS locations vs the number of dendrites in models having myelinated or unmyelinated axons. In all cases, increasing the number of dendrites led to more distal optimal AIS locations.

We next asked how somatodendritic morphology and AIS location interact to regulate neuron excitability by pairing the same somatodendritic architectures with a 30-µm-long AIS (8000 and 2000 pS/µm^2^ maximal sodium and potassium conductance densities, respectively) that could be positioned anywhere from 0 to 70 µm from the soma (Fig. 3). Regardless of whether the AIS was moved within a fixed 100 µm stretch of proximal axon (Fig. 1; AIS Model B; see Materials and Methods) or was connected to the soma by a variable-length proximal axon (Fig. 1; AIS Model C), optimal AIS location (i.e., that having the lowest rheobase current) was again dependent on somatodendritic size (Fig. 3*B*). In small neurons, rheobase currents were lowest when the AIS was positioned adjacent to the soma. However, as neurons increased in size, optimal AIS position moved to progressively more distal locations. This effect was largest when the AIS was associated with myelinated axons having low membrane capacitance and conductance, but also occurred when neurons were associated with unmyelinated axons (Fig. 3*B*, insets).

**Figure 4.**
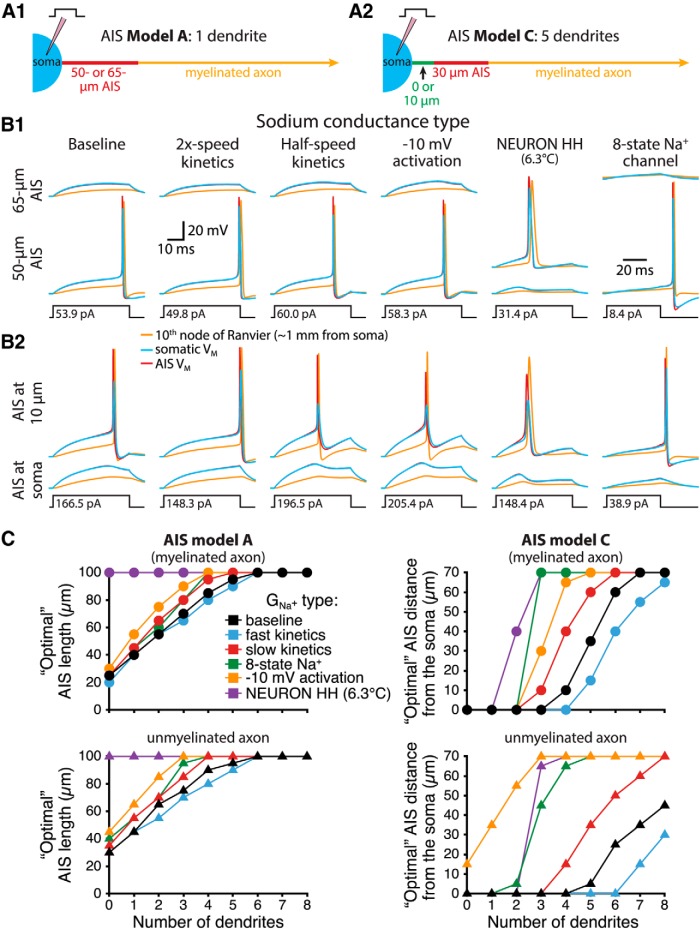
AIS performance across a range of sodium conductance properties. ***A***, Diagrams of the AIS of a one-dendrite myelinated neuron having a 50- or 65-µm-long AIS beginning at the soma (***A_1_***), and of a five-dendrite model with a 30-µm-long AIS beginning either at the soma or 10 µm away (***A_2_***). ***B***, Voltage responses at the soma (blue), AIS (red), or 10th node of Ranvier (∼1 mm from the soma; yellow) in response to the indicated current steps in models having the indicated sodium conductance properties and AIS architectures. In most one-dendrite models, increasing AIS length from 50 to 65 µm reduced neuron excitability (***B_1_***), while in the five-dendrite model, moving the 30-µm-long AIS 10 µm away from the soma increased neuron excitability (***B_2_***). ***C***, Comparisons of “optimal” (i.e., lowest rheobase) AIS lengths (left) and locations (right) for myelinated (top) and unmyelinated (bottom) model neurons having the indicated number of dendrites and sodium conductance properties.

Because excitability is critically dependent on the density and kinetics of sodium channels in the AIS, we compared the influence of somatodendritic morphologies on AIS performance in model neurons incorporating modified sodium conductances (Fig. 4). Modifications included doubling or halving the speed of activation and inactivation, lowering the voltage threshold for activation by 10 mV selectively in the AIS (as may result from selective sodium channel subunit expression in the AIS; Kole et al., 2008), implementation of an eight-state sodium channel model (Schmidt-Hieber and Bischofberger, 2010; Hallermann et al., 2012), or replacement of our standard active conductances with equivalent densities of the built-in Hodgkin and Huxley conductances in NEURON that simulate those in the squid giant axon (temperature in this implementation was set to 6.3°C). Modifications that slowed activation and inactivation tended to promote excitability in a longer or more distally located AIS, while increasing activation and inactivation rates favored a shorter or more proximally located AIS. Overall, the modified sodium conductances revealed qualitatively similar effects of somatodendritic morphology on AIS performance. The exception was for the NEURON Hodgkin and Huxley model, which when combined with the soma-only model neuron had “preferred” AIS lengths (i.e., lengths with lowest rheobase) beyond our normal test limit of 100 µm (Fig. 4*B*,*C*). Across all sodium conductances tested, rheobase in neurons having two or more dendrites decreased as a 30 µm AIS was moved distally from the soma (Fig. 4*C*). These data demonstrate that the impact of somatodendritic morphology on AIS performance is largely independent of the kinetics and voltage sensitivities of sodium channels.

**Figure 5.**
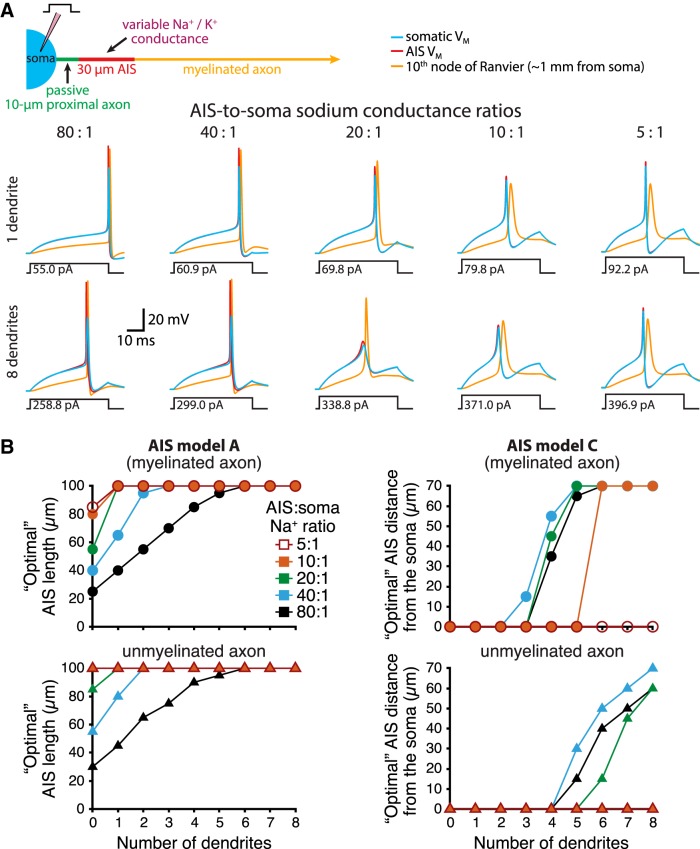
AIS performance across a range of AIS sodium conductance densities. ***A***, Top, Diagram of a model neuron having a 30-µm-long AIS positioned 10 µm from the soma. Bottom, Voltage responses measured at the soma (blue), in the AIS (red), or in the 10th node of Ranvier (∼1 mm from the soma; yellow) in response to the indicated rheobase current steps in models having the indicated number of dendrites and AIS-to-soma sodium conductance density ratios. ***B***, Comparisons of optimal (i.e., lowest rheobase) AIS lengths (left) and locations (right) for myelinated (top) and unmyelinated (bottom) model neurons having the indicated number of dendrites and AIS-to-soma sodium conductance densities.

We next varied the densities of sodium and potassium conductances in the AIS (normally 8000 and 2000 pS/µm^2^, respectively) to generate a range of AIS-to-soma sodium conductance ratios (Fig. 5). Across a span from our normal ratio of 80:1 to the lowest tested ratio of 5:1, somatodendritic morphologies continued to influence AIS performance, albeit to lesser extents as AIS-to-soma sodium conductance ratios became small (Fig. 5*B*,*C*). Overall, larger AIS conductance densities led to lowest rheobase currents occurring with shorter AIS lengths or more distal AIS locations.

**Figure 6.**
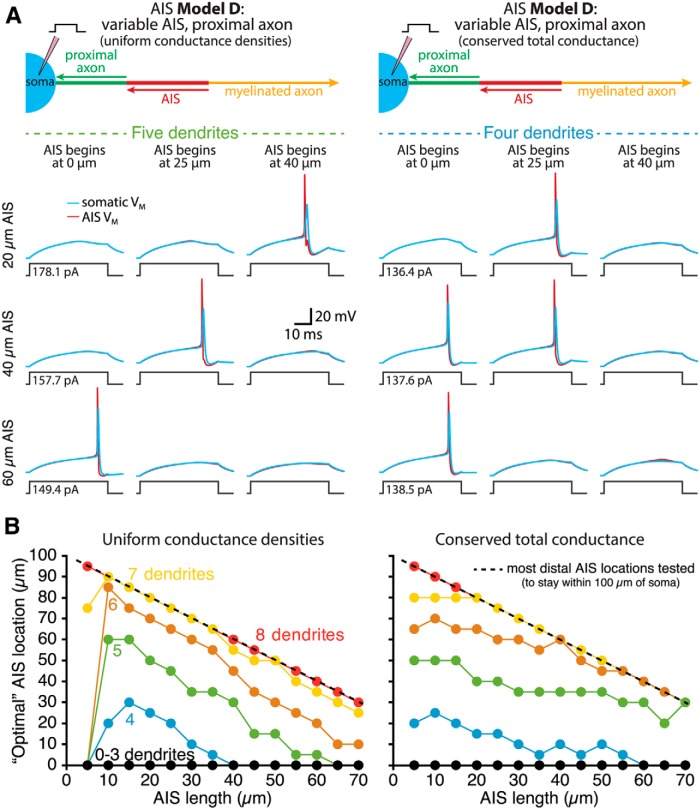
Interaction of AIS length and location in setting neuron excitability. ***A***, Diagrams (top) of the proximal axons of ball-and-stick neurons having variable-length AISs located at different distances from the soma (AIS Model D). The AISs had either uniform sodium and potassium conductance densities (i.e., sodium and potassium conductances increased with length; left) or conserved total sodium and potassium conductances (i.e., length-independent conductances; right). Voltage traces show somatic (blue) and AIS (red) responses to somatic current injections in model neurons having the indicated AIS lengths and locations. Note that in the 40-µm-long AIS model with conserved total conductances, rheobase (to the nearest 100 fA) was identical in models having the AIS beginning at 0 or 25 µm from the soma (minimal rheobase in this model occurred when the AIS was 10 µm from the soma). ***B***, Plots of optimal (i.e., lowest rheobase current) AIS position for each AIS length in models having “uniform conductance densities” (left) or “conserved total conductances” (right) in the AIS.

To test the interaction of AIS length and location in setting rheobase currents, we made hybrid models having a variable-length (5–70 µm long) AIS attached to a variable-length (0–95 µm long) soma-like proximal axon (AIS Model D). Regardless of whether the AIS contained uniform maximal conductance densities (Fig. 6, left) or conserved total conductances (Fig. 6, right), minimal rheobase currents in small neurons occurred when the AIS was positioned adjacent to the soma. On the other hand, larger neurons exhibited enhanced excitability when the AIS was located distally from the soma, and this distance-dependent enhancement of excitability was magnified when the AIS was short in length (Fig. 6*B*). The interaction of AIS length and location on rheobase currents can be visualized in rotating 3-D graphs showing data for neurons having zero, four, or eight dendrites, with the AIS having conserved active conductance densities (Movie 1) or conserved total active conductance magnitudes (Movie 2).

Movie 1. Interaction of AIS length and location in setting neuron excitability in model neurons having conserved conductance densities in the AIS. Shown is a rotating 3-D plot of normalized rheobase currents for each AIS length and location combination for the soma-only model neuron (blue), and neurons with four (green) or eight (red) dendrites in models where the AIS has conserved conductance densities (i.e., total available sodium and potassium conductances increase with AIS length). 10.1523/ENEURO.0085-15.2016.video.1

Movie 2. Interaction of AIS length and location in setting neuron excitability in model neurons having conserved total AIS conductances. Shown is a rotating 3-D plot of normalized rheobase currents for each AIS length and location combination for the soma-only model neuron (blue), and neurons with four (green) or eight (red) dendrites in models having conserved (i.e., length-independent) maximal sodium and potassium conductances in the AIS. 10.1523/ENEURO.0085-15.2016.video.2

**Figure 7.**
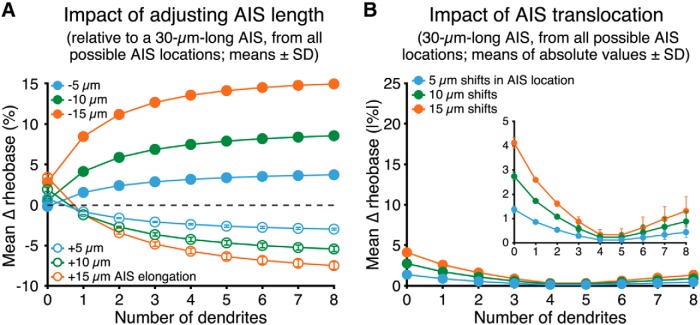
Impact of AIS plasticity on neuron excitability. ***A***, Plots of mean changes in rheobase currents (percentage ±SD) observed after elongating or shortening a 30-µm-long AIS by 5, 10, or 15 µm across all possible AIS locations (within 100 µm of the soma) in model neurons having the indicated number of dendrites (AIS Model D; myelinated axon). Uniform sodium and potassium conductance densities were maintained across all AIS lengths (i.e., increasing AIS length proportionally increased total maximum sodium and potassium conductances). ***B***, Plots of the mean absolute values of changes in rheobase (percentage ±SD) observed after translocating a 30-µm-long AIS by 5, 10, or 15 µm across all possible AIS positions within 100 µm of the soma (e.g., 14 possible 5 µm shifts of the 30 µm AIS) in neurons having the indicated number of dendrites (AIS Model C). To facilitate comparisons, data are shown at the same scale as in ***A***, with a close-up of the data inset.

To quantify the impact of AIS plasticity on neuron excitability, we measured the average change in rheobase current generated by small (5, 10, or 15 µm) changes in AIS length (from a baseline length of 30 µm, while maintaining uniform conductance densities), or position (using a 30-µm-long AIS), across all possible AIS locations within 100 µm of the soma. Except for the soma-only model (see Fig. 2*B*), lengthening or shortening the AIS increased or decreased, respectively, neuron excitability. However, the magnitude of this change in excitability was dependent on somatodendritic morphology (Fig. 7*A*). In the soma-only model, increases or decreases in AIS length by up to 50% (i.e., adding or subtracting 15 µm to or from the 30 µm AIS) resulted in small (<5%) changes in excitability. In larger neurons, increasing AIS length led to ever-lower rheobase currents, with a maximal reduction of 7.5 ± 0.6% (*n* = 12 possible AIS locations allowing for lengthening of 15 µm confined within 100 µm of the soma) following 50% elongations (i.e., to 45 µm) in the eight-dendrite model (Fig. 7*A*). Conversely, decreasing AIS length by 50% (i.e., to a length of 15 µm) increased rheobase currents by up to 15.0 ± 0.4% (*n* = 15) in the eight-dendrite model (Fig. 7*A*).

Changes in AIS location were far less effective in regulating neuron excitability. Across all somatodendritic morphologies, translocations of the 30-µm AIS by up to ±15 µm produced only small changes in rheobase currents (Fig. 7*B*), with the magnitude and direction of those changes being dependent on neuron size (Fig. 3*B*). Mean changes in rheobase currents (absolute values) generated by translocations of 15 µm across all possible AIS locations within 100 µm of the soma ranged from a high of 4.1 ± 0.2% (*n* = 12 possible 15-µm shifts) in the soma-only model, to a low of 0.3 ± 0.3% (*n* = 12) in the five-dendrite model neuron (Fig. 7*B*). These results suggest that AIS translocations of up to 15 µm will have only limited impact on neuron excitability across a range of neuron morphologies.

### AIS performance in realistic neuron morphologies

**Figure 8.**
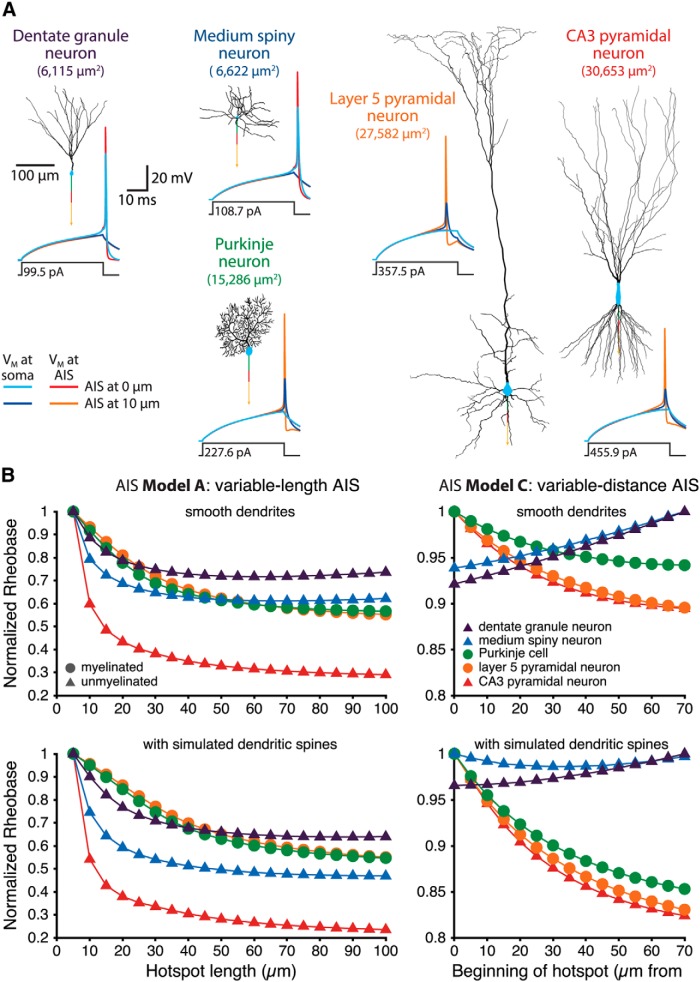
AIS performance in morphologically realistic neurons. ***A***, Morphologies of reconstructed neurons. For illustrative purposes, each morphology includes a 40-µm-long proximal axon (green), a 30-µm-long AIS (red), and a myelinated (in the Purkinje and L5 neurons) or unmyelinated (in the medium spiny, dentate granule, and CA3 pyramidal neurons) axon (yellow arrow). For each morphology, somatic and AIS voltage traces in response to the indicated somatic current injections are shown for models having a 30 µm AIS located adjacent to the soma or 10 µm away. Note that excitability in the smaller neurons is promoted by proximal AIS placement, but that the larger neurons favor a more distal AIS. ***B***, Left panels, Plots of normalized rheobase current vs AIS length (AIS Model A; uniform sodium and potassium conductance densities) for reconstructed neurons having smooth dendrites (top) or simulated synaptic spines (bottom; see Materials and Methods). Smaller neurons exhibited minimal rheobase currents at intermediate AIS lengths, while the simulation of dendritic spines favored an elongated AIS. Right panels, Plots of normalized rheobase current vs location of a 30-µm-long AIS (AIS Model C) in reconstructed neurons having smooth (top) or simulated spiny (bottom) dendrites. Smaller neurons favored a proximal AIS, while simulating spiny dendrites shifted optimal AIS locations away from the soma.

We next tested the impact of AIS length and location on rheobase currents in five reconstructed neuron morphologies (Fig. 8*A*). Neurons ranged from a small dentate granule neuron (total somatodendritic membrane area, 6115 µm^2^) to a large CA3 pyramidal neuron (total somatodendritic membrane area, 30,653 µm^2^). AIS segments were attached to myelinated (Purkinje and L5 neurons) or unmyelinated (dentate granule, medium spiny, and CA3 neurons) axons, as appropriate for their cell types, and rheobase currents determined to the nearest 100 fA using somatic current injections (40 ms).

When morphologically realistic model neurons were paired with a variable-length AIS having uniform maximal sodium and potassium conductances (AIS Model A), the smaller dentate granule and medium spiny neurons exhibited minimal rheobase currents when the AIS was of intermediate length (60 and 65 µm, respectively), while larger neurons had the lowest rheobase currents when AIS length reached its maximum tested extent (100 µm; Fig. 8*B*, top left). When dendritic spines were simulated by doubling dendritic membrane capacitance and passive conductance (see Materials and Methods; Holmes, 1989), optimal AIS lengths in the dentate granule and medium spiny neurons increased to 85 and 100 µm, respectively (Fig. 8*B*, bottom left).

Similarly, when a 30-µm-long AIS was positioned at variable distances (0-70 µm) from the soma (AIS Model C), rheobase currents in smaller neurons were minimal when the AIS was adjacent to the soma, while larger neurons were most excitable when the AIS was moved to distal locations (Fig. 8*A*, traces, *B*, top right panel). Simulation of dendritic spines further favored distal AIS locations, with rheobase in the medium spiny neuron becoming lowest when the AIS was positioned 30 µm from the soma (Fig. 8*B*, bottom right).

**Figure 9.**
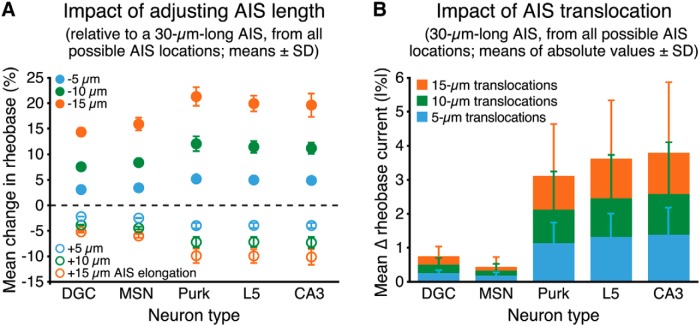
Impact of AIS plasticity on the excitability of morphologically realistic neurons. ***A***, Plots of the mean changes in rheobase currents (percentage ±SD) observed after elongating or shortening the AIS by 5, 10, or 15 µm (relative to a 30-µm-long baseline AIS), across all possible AIS locations within 100 µm of the soma, in reconstructed neuron morphologies having simulated dendritic spines (see Materials and Methods) and uniform sodium and potassium conductance densities in the AIS (AIS Model D). ***B***, Plots of the mean absolute values of changes in rheobase (percentage ±SD) observed after translocating the AIS by 5, 10, or 15 µm across all possible AIS positions within 100 µm of the soma. Note the different *y*-axis scales in ***A*** and ***B***.

We quantified the impact of changes in AIS length and location on the excitability of these morphologically realistic neurons by measuring the mean changes in rheobase current generated by small (5-15 µm) changes in AIS length or location (relative to a baseline 30-µm-long AIS) across all possible AIS locations up to 100 µm from the soma (Fig. 9). In all neuron morphologies, lengthening or shortening the AIS decreased or increased, respectively, rheobase currents (Fig. 9*A*). Lengthening the AIS by 50% (i.e., by 15 µm) lowered rheobase currents by a minimum of 5.2 ± 0.3% (in the dentate granule neuron) to a maximum of 10.2 ± 1.7% (in the CA3 pyramidal neuron; *n* = 12 possible AIS locations for each neuron), while shortening the AIS by 50% (i.e., by 15 µm) increased rheobase currents by 14.3 ± 1.0% (dentate granule cell) to 21.3 ± 2.0% (Purkinje neuron; Fig. 9*A*). To further demonstrate the interaction of somatodendritic morphology and AIS length in regulating neuron excitability, we compared action potential generation in response to suprathreshold current injections in two versions of the dentate granule neuron (Movie 3) and L5 pyramidal neuron (Movie 4) that differed only in their AIS lengths. As illustrated in the movies, action potential initiation in the dentate granule neuron occurred first when the AIS was shorter, while spike initiation in the L5 neuron occurred earlier when the AIS was longer.

Movie 3. Impact of AIS length on action potential initiation in a dentate granule neuron. Shown are two superimposed plots of membrane voltage (in millivolts) vs compartment distance (in micrometers) from the soma, spanning the axon (positive distances from the soma) to the longest dendrite (negative distances from the soma), for two versions of the dentate granule neuron having either a 60-µm-long (red) or an 80-µm-long (green) AIS beginning adjacent to the soma. Current (100 pA) was injected into the soma of each model beginning at time = 2 ms. Note that the timescale slows down to show initiation in high temporal resolution, which occurs first in the DGC with the shorter AIS. 10.1523/ENEURO.0085-15.2016.video.3

Movie 4. Impact of AIS length on action potential initiation in a layer 5 pyramidal neuron. Shown are two superimposed plots of membrane voltage (in millivolts) vs compartment distance (in micrometers) from the soma, spanning the axon (positive distances from the soma) to the longest dendrite (negative distances from the soma) for two versions of the L5 pyramidal neuron that have either a 60-µm-long (red) or an 80-µm-long (green) AIS beginning adjacent to the soma. Current (757 pA) was injected into the soma of each model beginning at time = 2 ms. Note that the timescale slows down to show initiation in high temporal resolution, which occurs first in the L5 neuron with the longer AIS. 10.1523/ENEURO.0085-15.2016.video.4

We next quantified the impact of changes in AIS location on action potential threshold in morphologically realistic neurons. AIS translocations of up to ±15 µm produced only small (<5%; absolute values) changes in rheobase currents across all somatodendritic morphologies (Fig. 9*B*), with the magnitude of those changes being dependent on neuron size, and the direction of change depending on both neuron size and starting AIS position. Overall, mean changes in rheobase currents (absolute values) generated by 15-µm shifts at all possible AIS locations within 100 µm from the soma were small, ranging from a low of 0.4 ± 0.3% (*n* = 12 possible 15-µm shifts) in the medium spiny neuron to a high of 3.8 ± 2.1% (*n* = 12) in the CA3 model neuron (Fig. 9*B*). To visualize the interaction of somatodendritic morphology and AIS location in regulating neuron excitability, we again compared action potential initiation in the dentate granule neuron (Movie 5) and the L5 pyramidal neuron (Movie 6) in response to suprathreshold somatic current current injections in models where the AIS was positioned adjacent to the soma or 15 µm away. In the smaller dentate granule neuron, action potential initiation occurred earlier when the AIS was adjacent to the soma, while spike initiation occurred first in the L5 neuron when the AIS was moved away from the soma. Together, these data from morphologically realistic neurons reveal a profound influence of somatodendritic morphology on the outcome of plastic change in the AIS, and further demonstrate that changes in AIS length will be far more effective at regulating neuron excitability than will similar changes in AIS location.

Movie 5. Impact of AIS location on action potential initiation in a dentate granule neuron. Shown are two superimposed plots of membrane voltage (in millivolts) vs compartment distance (in micrometers) from the soma, spanning the axon (positive distances from the soma) to the longest dendrite (negative distances from the soma) for two versions of the dentate granule neuron associated with a 30-µm-long AIS positioned either adjacent to these soma (red) or 15 µm from the soma (green). Current (120 pA) was injected into the soma of each model at time = 2 ms. Note that the timescale slows down to show initiation in high temporal resolution, which occurs first when the AIS is adjacent to the soma. 10.1523/ENEURO.0085-15.2016.video.5

Movie 6. Impact of AIS location on action potential initiation in a layer 5 pyramidal neuron. Shown are two superimposed plots of membrane voltage (in millivolts) vs compartment distance (in micrometers) from the soma, spanning the axon (positive distances from the soma) to the longest dendrite (negative distances from the soma) for two versions of the L5 pyramidal neuron associated with a 30-µm-long AIS positioned either adjacent to these soma (red) or 15 µm from the soma (green). Current (757 pA) was injected into the soma of each model at time = 2 ms. Note that the timescale slows down to show initiation in high temporal resolution, which occurs first when the AIS is distal from the soma. 10.1523/ENEURO.0085-15.2016.video.6

Why do manipulations of AIS length or location in morphologically realistic neurons impact excitability in ways not predictable based purely on neuron size? For instance, the largest effects of AIS shortening were observed in the mid-sized Purkinje neuron (Fig. 9*A*). This likely results from several features of our realistic neuron morphologies. First, the Purkinje neuron and the layer 5 neuron were paired with myelinated axons, which enhanced the impact of neuron size on AIS performance in our ball-and-stick models. Second, the realistic morphologies were relatively large; the “small” dentate granule (6115 µm^2^) and medium spiny (6622 µm^2^) neurons were of sizes intermediate to the three-dendrite (5498 µm^2^) and four-dendrite (6912 µm^2^) ball-and-stick model neurons, while all other realistic morphologies were larger than our eight-dendrite ball-and-stick neuron (12,566 µm^2^). There may be a ceiling effect limiting additional impact of very large surface areas on optimal AIS length (Fig. 2*B*). Finally, morphologically realistic neurons have unique and complicated dendritic branching patterns that distribute surface area unevenly with respect to the soma (e.g., much of the dendritic tree in the L5 pyramidal neuron is in the distal tuft, remote from the soma and AIS). Differences in dendritic proximity to the axon will likely influence AIS performance (van Ooyen et al., 2002; van Elburg and van Ooyen, 2010).

**Figure 10.**
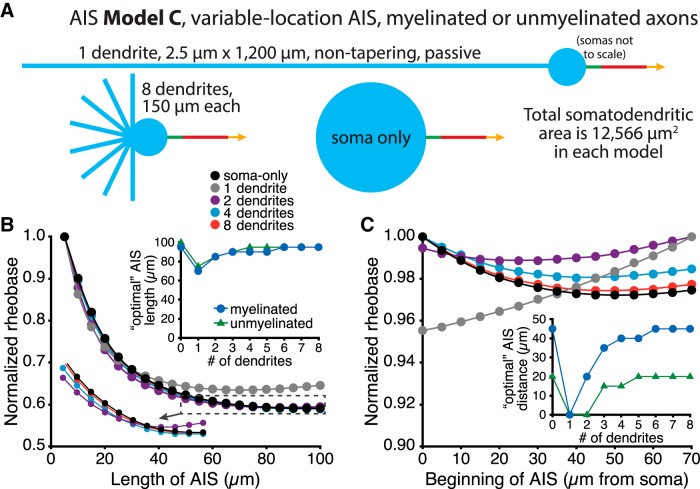
Impact of dendritic topology on AIS performance. ***A***, Diagrams of ball-and-stick model neurons (AIS Model C) having zero, one, or eight nontapering (2.5 µm diameter) passive dendrites that conserve a total somatodendritic surface area of 12,566 µm^2^ in each model. In the soma-only model, the densities of active somatic conductances were reduced in the enlarged soma to conserve the same total conductances present in the baseline soma. ***B***, Plots of normalized rheobase currents for different AIS lengths (AIS Model A; conserved sodium and potassium conductance densities) in model neurons having variable somatodendritic topology, but conserved total surface areas. Top inset, Plots of optimal AIS length vs the number of dendrites in models having preserved total somatodendritic surface area. Bottom inset, An expanded view of the indicated data points. ***C***, Plots of normalized rheobase currents for the same model neurons having a 30-µm-long AIS placed at the indicated distances from the soma (AIS Model C). Inset, Plots of optimal AIS distance from the soma vs the number of dendrites in models having preserved total somatodendritic surface area.

To test the impact of somatodendritic topology on AIS performance, we built a series of ball-and-stick models (AIS Model C) with zero to eight passive dendrites that conserved total somatodendritic surface area (12,566 µm^2^; Fig. 10*A*) and compared the impact of changes in AIS length (Fig. 10*B*) or location (Fig. 10*C*) on rheobase currents. Under these conditions, where dendritic topology alone was manipulated, models in which dendrites were more numerous but shorter in length (i.e,. models in which dendritic surface area was closer to the soma) favored longer AIS lengths and more distal AIS locations, and this effect was maximal when dendrites were eliminated altogether and the soma enlarged to match the total somatodendritic surface area of the other models. The impact of dendritic topology on optimal AIS length and location was qualitatively similar in models with myelinated and unmyelinated axons (Fig. 10*B*,*C*, insets) and had an overall larger influence on optimal AIS location than on optimal AIS length (Fig. 10*B*,*C*). These data demonstrate that the impact of AIS plasticity on neuronal excitability will depend not only on overall somatodendritic size, but also on dendritic topology.

### Dendritic membrane properties influence optimal AIS location

**Figure 11.**
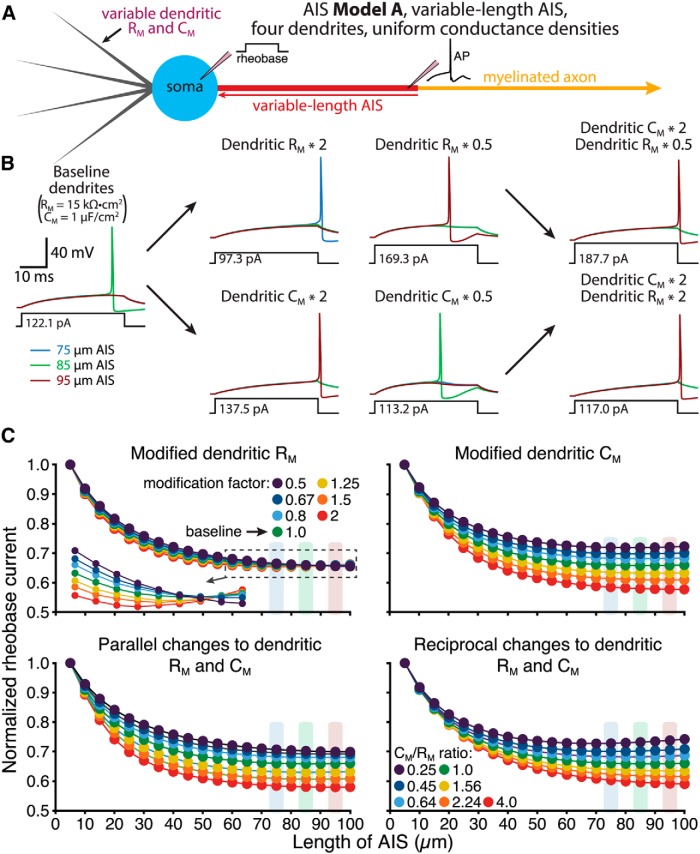
Impact of dendritic membrane properties on optimal AIS length. ***A***, Diagram of a neuron with four dendrites having variable dendritic membrane properties and a variable-length AIS attached to a myelinated axon (AIS Model A). ***B***, Voltage responses in the AIS following somatic current injections under baseline conditions (far left) and after manipulations of dendritic R_M_ and/or C_M_, as indicated. Responses from three model neurons having AIS lengths of 75 (blue), 85 (green), and 95 (red) µm are superimposed. ***C***, Plots of normalized rheobase current vs AIS length for the four dendrite model having modified dendritic R_M_ (top left; inset shows close-up of indicated area), dendritic C_M_ (top right), parallel changes in both dendritic R_M_ and C_M_ (bottom left), or reciprocal changes in dendritic R_M_ and C_M_ (bottom right). Dendritic C_M_ and/or R_M_ were multiplied by the indicated modification factors. In experiments testing reciprocal changes in dendritic properties (bottom right), the C_M_/R_M_ ratios represent reciprocal changes to C_M_ and R_M_ around the baseline (C_M_/R_M_ ratio = 1) values (e.g., a ratio of 0.25 indicates that dendritic C_M_ was multiplied by 0.5 and dendritic R_M_ was multiplied by 2.0). Increasing dendritic C_M_ favored a longer AIS, regardless of whether there were also co-occurring changes in dendritic conductance (i.e., changes in dendritic R_M_). Shaded bars indicate the AIS lengths for which traces are shown in ***B***.

The results described above demonstrate that somatodendritic morphology will impact AIS plasticity, especially with regard to changes in AIS location. Given that neuron size primarily influences somatodendritic capacitance and conductance, we hypothesized that changes to these dendritic membrane properties should be sufficient to regulate optimal AIS architecture, even in the absence of structural changes to neuron morphology. To test this, we varied dendritic capacitance (by scaling dendritic C_M_) and/or conductance (by scaling dendritic R_M_) in a four-dendrite ball-and-stick model neuron and measured rheobase currents across a range of AIS architectures (Fig. 11). Overall, increases in dendritic conductance (i.e., decreases in dendritic R_M_) or capacitance favored longer optimal AIS lengths (Fig. 11*B*,*C*), although changes to dendritic C_M_ had a larger impact on AIS performance than did changes in dendritic R_M_ (Fig. 11*C*).

**Figure 12.**
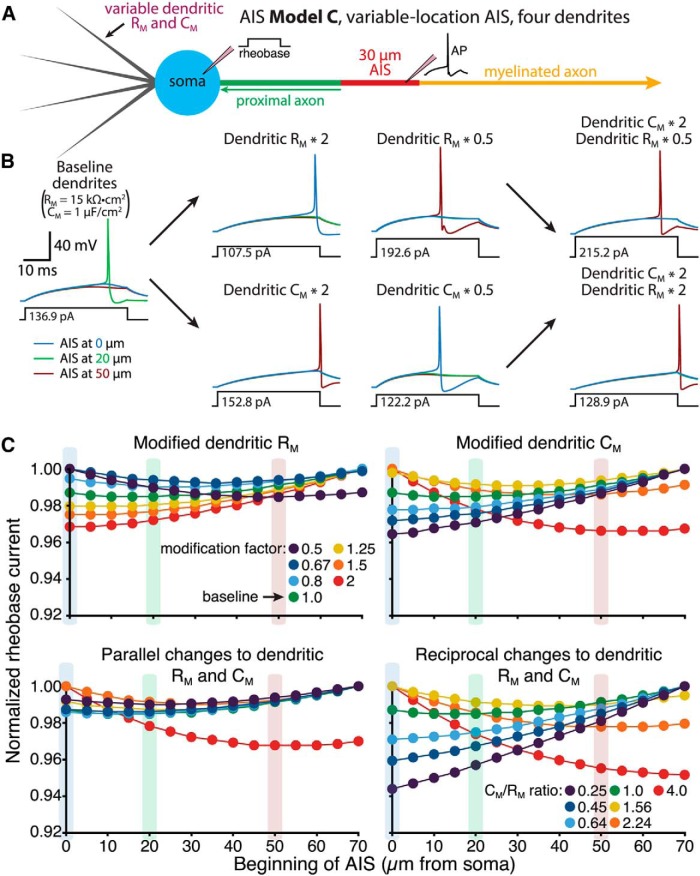
Dendritic membrane properties influence optimal AIS location relative to the soma. ***A***, Diagram of a neuron with four dendrites having variable dendritic membrane properties and a 30 µm AIS positioned at variable distances from the soma (AIS Model C; myelinated axon). ***B***, Voltage responses in the AIS during somatic current injections under baseline conditions (far left) and after manipulations of dendritic R_M_ and/or C_M_, as indicated. Responses from three models are superimposed: those having the AIS beginning 0 (blue), 20 (green), or 50 µm (red) from the soma. ***C***, Plots of normalized rheobase current vs AIS location for the four-dendrite models having modified dendritic R_M_ (top left), dendritic C_M_ (top right), parallel changes in both dendritic R_M_ and C_M_ (bottom left), or reciprocal changes in dendritic properties (bottom right). Dendritic C_M_ and/or R_M_ were multiplied by the indicated modification factors. C_M_/R_M_ ratios in reciprocal manipulations of R_M_ and C_M_ are centered around baseline (C_M_/R_M_ ratio = 1) values. Increasing dendritic C_M_ and/or dendritic conductance (lower dendritic R_M_) enhanced excitability at more distal AIS locations. Shaded bars indicate the AIS locations for which traces are shown in ***B***.

We next tested the impact of manipulations of dendritic R_M_ and C_M_ on excitability in a four-dendrite neuron having a 30-µm-long AIS connected to a myelinated axon and positioned at variable distances from the soma (AIS Model C; Fig. 12*A*). In baseline conditions (i.e., dendritic C_M_ and R_M_ set to 1 µF/µm^2^ and 15,000 Ω · cm^2^, respectively), minimal rheobase currents occurred when the AIS was positioned 10 µm from the soma. Increasing dendritic C_M_, or decreasing dendritic R_M_ (i.e., increasing dendritic membrane conductance), shifted optimal AIS locations away from the soma (Fig. 12*B*,*C*). On the other hand, decreasing dendritic C_M_, or increasing dendritic R_M_, favored more proximal AIS locations. Changes to dendritic C_M_ had a larger impact on optimal AIS location, as doubling both dendritic C_M_ and R_M_ continued to favor more distal AIS locations (to almost the same extent as doubling dendritic C_M_ alone), while halving both values had minimal impact on preferred AIS location (Fig. 12*C*, bottom left traces). Reciprocal changes in dendritic R_M_ and C_M_ were most effective in moving optimal AIS locations (Fig. 12*C*, bottom right traces). Qualitatively similar effects of dendritic R_M_ and C_M_ on excitability were observed in model neurons incorporating the modified sodium conductances used in Fig. 4 (not shown), suggesting that dendritic membrane properties have a generalized impact on AIS performance.

### Action potential initiation in the AIS

**Figure 13.**
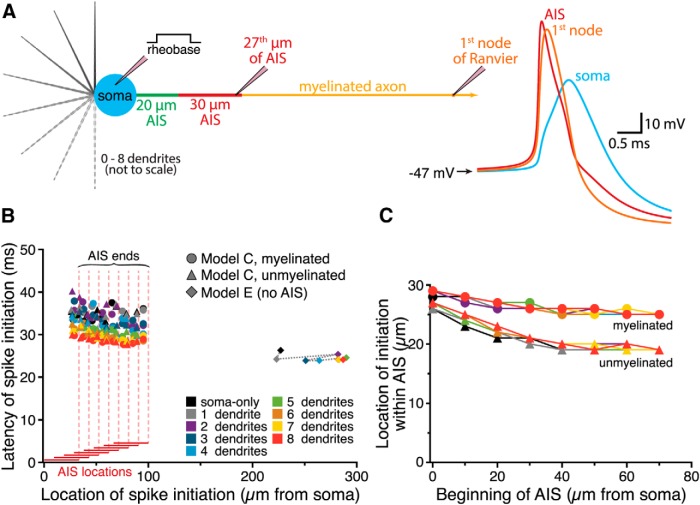
Dendritic architecture influences action potential initiation. ***A***, Left, Diagram of a model neuron having zero to eight dendrites, a 30 µm AIS located 20 µm from the soma, and a myelinated axon. Pipettes indicate the location of recording electrodes at the soma (where rheobase current is injected), toward the end of the AIS (where action potential initiation occurred) and at the first node of Ranvier. Right, Traces of the action potential generated by rheobase somatic current injection, as recorded at the indicated locations in the model cell. These simulations were run with 100 ns time steps to pinpoint action potential initiation to the nearest 1 µm. ***B***, Plot of spike latency vs location of spike initiation for myelinated and unmyelinated neurons with different numbers of dendrites (as indicated by color) and variable AIS locations (AIS Model C), and for neurons having uniform unmyelinated axons lacking an AIS (AIS Model E). The AIS constrained the location of action potential initiation, but increased the influence of dendrite number on spike latency. ***C***, Plot of the location of action potential initiation within the AIS for myelinated and unmyelinated neurons vs the distance of the AIS from the soma. When the AIS was placed at distal locations, action potential initiation occurred in slightly more proximal AIS compartments, an effect that was more pronounced in neurons with unmyelinated axons.

Action potential initiation has been observed to occur first in the distal AIS (Palmer and Stuart, 2006; Palmer et al., 2010), likely due to its relative isolation from somatodendritic capacitive loads (Baranauskas et al., 2013). To determine the extent to which somatodendritic architecture influences the timing and location of action potential initiation, we compared the location and latency of rheobase-induced action potentials in simulations run at 100 ns time steps (Fig. 13). Myelinated and unmyelinated model neurons included variable numbers of dendrites and AIS locations (AIS Model C). For comparison, we also tested an unmyelinated model neuron lacking an AIS altogether (AIS Model E). Latency to action potential initiation was influenced primarily by neuron size, with larger neurons firing rheobase action potentials earlier than smaller neurons, and by AIS location, where shorter-latency action potentials were promoted at distal AIS positions (Fig. 13*B*). When an AIS was present, action potential initiation always occurred at the distal end of the AIS, with the precise location of initiation being dependent on AIS distance from the soma, but not on somatodendritic size (Fig. 13*C*). Conversely, action potential generation in a neuron lacking an AIS occurred at very distal locations (>200 µm from the soma; Fig. 13*B*).

**Figure 14.**
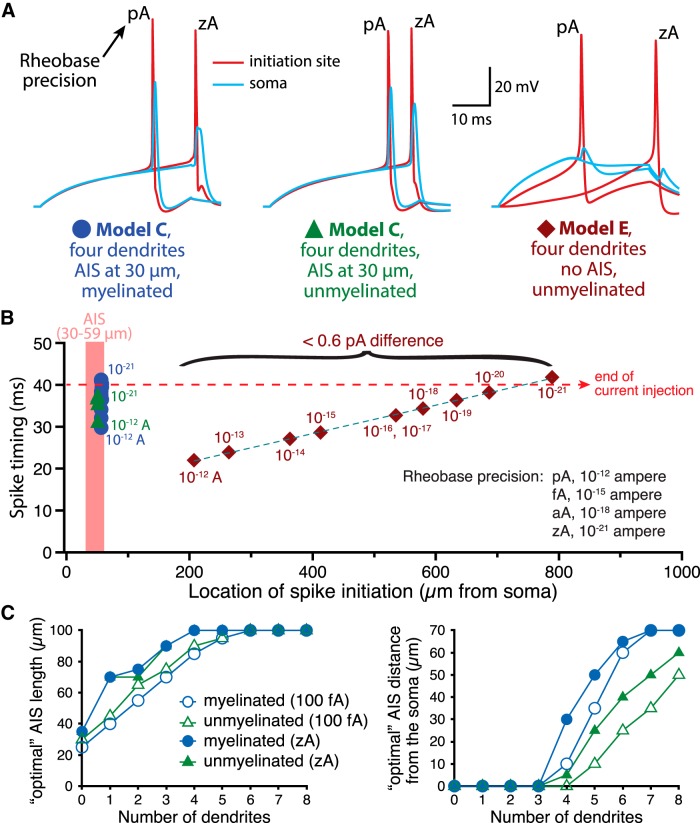
Impact of rheobase precision on action potential initiation and AIS plasticity. ***A***, Plots of action potential waveforms recorded in the AIS (red; 30 µm long and 30 µm from the soma) or in the soma (blue) of neurons with four dendrites and either an AIS associated with a myelinated axon (left), an unmyelinated (middle) axon (AIS Model C), or an unmyelinated axon lacking an AIS (AIS Model E; right). Shown are traces in which rheobase current was calculated to the nearest pA or to the nearest zA, as indicated (simulations used 100 ns time-steps). ***B***, Plots of spike latency vs the location of spike initiation in myelinated and unmyelinated neurons having an AIS (Model C), or in a neuron lacking an AIS (Model E), for rheobase current injections at the indicated precision levels (i.e, rounded up to the nearest suprathreshold stated unit). Note that approaching ever more precise rheobase currents increased the latency to spike initiation and, in the absence of an AIS, moved the location of initiation to very distal regions of the axon. ***C***, Plots of AIS lengths (left) or locations (right) having the lowest rheobase currents, as determined to the nearest 100 fA (open symbols) or 1 zA (closed symbols) in myelinated and unmyelinated neurons.

In the simulations above, in which rheobase was determined to the nearest 100 fA, we observed jitter in action potential location and timing due to rounding errors (i.e., the degree to which rheobase determined to the nearest 100 fA exceeded action potential threshold for any given morphology), especially in models lacking an AIS. To explore action potential initiation at more precise values for rheobase, we determined rheobase at ever more accurate levels, from the nearest 1 pA to to the nearest 1 zeptoampere (zA; 10^−21^ A; Fig. 14). Increasing the precision of rheobase currents increased spike latency in all models and, in models lacking an AIS, moved action potential initiation to ever more distal axonal locations, up to many hundreds of micrometers from the soma (Fig. 14*B*), sites that in many neurons would be well beyond proximally located synaptic boutons. These data suggest that one advantage of having an AIS may be greater precision in action potential initiation timing and location. Nevertheless, increasing rheobase precision did not qualitatively change the impact of somatodendritic morphology on AIS plasticity in myelinated or unmyelinated neurons (Fig. 14*C*), suggesting that the impact of AIS plasticity is independent of the timing of action potential initiation relative to current onset.

### Somatodendritic morphology shapes electrical properties of the AIS

**Figure 15.**
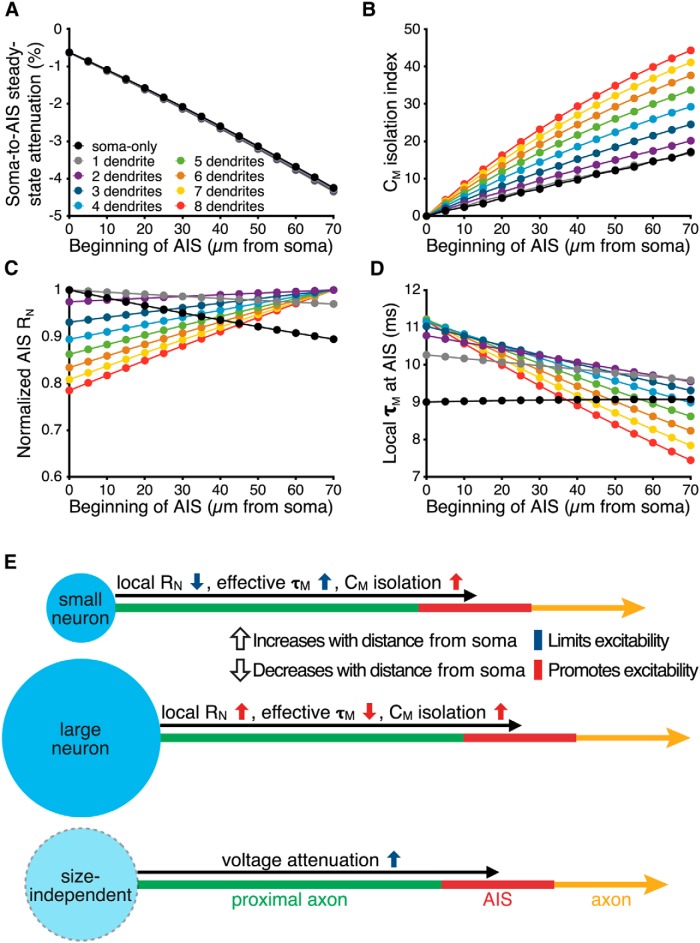
Mechanisms controlling the impact AIS plasticity. ***A***, Steady-state voltage attenuation, measured in the middle of a 30-µm-long AIS positioned at the indicated distances from the soma (AIS Model C) in response to a somatic current injection (−1 pA) delivered at the resting membrane potential. Data are from model neurons having zero (black symbols) to eight (red symbols) dendrites. ***B***, C_M_-isolation index (see Methods) vs AIS distance from the soma for neurons having zero to eight dendrites. ***C***, Normalized local steady-state input resistance (R_N_) vs AIS location. ***D***, Local effective membrane time constant (τ_M_) in the AIS vs distance from the soma. ***E***, Summary diagrams of location-dependent changes in the biophysical properties of the AIS in neurons of different sizes. Arrows indicated the direction of change in the indicated biophysical property as the AIS moves distally away from the soma. Arrow colors indicate whether such changes will limit (blue) or enhance (red) neuron excitability.

Why are larger neurons more excitable when the AIS is moved to distal locations? To address this larger question, we tested AIS electrical characteristics in model neurons in which a 30-µm-long AIS was connected to a myelinated axon and positioned at variable distances from the soma (AIS Model C). Since distance-dependent attenuation of somatic depolarization is proposed to limit excitation when the AIS is located distally from the soma (Adachi et al., 2014), we compared voltage attenuation of somatic signals at the middle of the AIS across all models. In response to a −1 pA current injection at the soma (100 ms), distance-dependent voltage attenuation to the AIS was small, with mean attenuation in the middle of an AIS positioned at the maximally tested distance of 70 µm from the soma being only 4.32 ± 0.03% (*n* = 9 somatodendritic morphologies), and independent of neuron size (Fig. 15*A*). This result is in good agreement with experimental data from Kole et al. (2007), who found only modest soma-to-AIS voltage attenuation (<10%) within the first 150 µm of the axon.

Although modest, distance-dependent voltage attenuation would, on its own, favor lower rheobase currents at proximal AIS locations. However, distal locations also isolate the AIS from somatodendritic capacitive loads (Kuba, 2012). We quantified distance-dependent isolation from somatodendritic capacitance by calculating a C_M_ isolation index for each AIS location (see Materials and Methods; Fig. 15*B*). The C_M_ isolation index increased with distance in all morphologies, but exhibited a steeper relationship with distance in larger neurons, from a 17% reduction in the influence of somatodendritic capacitance in the soma-only model when the AIS was positioned 70 µm from the soma, to a 44% reduction in a similar model having eight dendrites. This distance-dependent isolation from somatodendritic capacitance will tend to facilitate action potential generation at more distal AIS locations, especially in larger neurons.

Action potential initiation is directly influenced by the magnitude and speed of local membrane depolarization in the AIS (Baranauskas et al., 2013; Eyal et al., 2014), and therefore the local input resistance (R_N_) and effective membrane time constant (τ_M_) of the AIS will influence rheobase currents. We therefore measured R_N_ (Fig. 15*C*) and τ_M_ (Fig. 15*D*) at the middle of the AIS in response to local current injections (−1 pA) delivered at resting membrane potentials. In small neurons (those with fewer than two dendrites), local R_N_ in the AIS decreased with distance from the soma (by 10.7% in the soma-only model), while in larger neurons local R_N_ increased with distance, exhibiting up to a 27.4% increase over 70 µm in the eight-dendrite model neuron. On the other hand, the effective local τ_M_, calculated empirically by measuring the latency at which the local voltage response reached 63.2% of its steady-state value (Fig. 15*D*), increased negligibly with AIS distance in the soma-only model (by 0.5% over 70 µm), but decreased with distance in larger neurons, by as much as 33.2% over 70 µm in the eight-dendrite model neuron (Fig. 15*D*). The impact of these physiological properties on AIS performance is summarized in Fig. 15*E*. When remeasured after moving the reversal potential of leak conductances closer to the action potential threshold (from −70 to −55 mV), maximum soma-to-AIS voltage attenuation remained at <5% for all models, and the impact of somatodendritic architecture on local R_N_, effective τ_M_, and isolation from somatodendritic capacitance was qualitatively similar to that measured at −70 mV (data not shown). Thus, while modest soma-to-AIS voltage attenuation will tend to decrease excitability in all neurons, local properties promoting action potential generation (higher R_N_, faster effective τ_M_, and more effective isolation from somatodendritic capacitance) are more pronounced at distal AIS locations in larger neurons and, therefore, allow rheobase currents to decrease, rather than increase, as the AIS is lengthened or translocated away from the soma.

## Discussion

The AIS represents the final locus of synaptic integration and, therefore, is an ideal target for regulating action potential initiation. Indeed, the AIS is often a site of inhibitory synaptic input, and is enriched with a diverse range of voltage- and ligand-gated ion channels that can promote or suppress action potential generation (for review, see Kole and Stuart, 2012). Several recent studies have described a novel intrinsic mechanism for homeostatic regulation of neuronal excitability: dynamic structural remodeling of the AIS in response to altered excitatory drive (Grubb and Burrone, 2010; Kuba et al., 2010; Muir and Kittler, 2014; Chand et al., 2015). These structural modifications, which include changes to AIS length and/or location, are hypothesized to stabilize the mean level of output of a neuron following chronic changes in synaptic drive (Grubb et al., 2011). Our simulations tested the impact of AIS architecture on intrinsic neuronal excitability across a wide range of somatodendritic morphologies. Our results demonstrate that somatodendritic morphology will have a profound impact on AIS performance and plasticity.

### Impact of somatodendritic morphology on AIS plasticity

Using a wide range of neuron morphologies and sodium conductance properties, we found that smaller neurons tended to be most excitable when the AIS was of intermediate length and located adjacent to the soma, while excitability in larger neurons was enhanced when the AIS was longer and more distally located. Modifications of AIS length were far more effective in regulating neuron excitability, and less dependent on somatodendritic morphology, than were changes in AIS location. While AIS elongations of 15 µm were capable of increasing neuron excitability by up to ∼20%, AIS translocations of 15 µm had only modest impact on excitability (≤4% change), with the direction of change being dependent on somatodendritic morphology and AIS starting position within the axon. Further, because distal translocations of the AIS can limit action potential propagation into somata and dendrites (Fig. 3*A*), such plasticity may prove maladaptive to neurons that depend upon backpropagating action potentials for normal synaptic integration or plasticity (Bi and Poo, 1998; Larkum et al., 1999). Overall, the impact of neuron size on AIS performance was similar in both myelinated and unmyelinated neurons, with unmyelinated neurons of a given size acting like somewhat smaller myelinated neurons in their response to changes in AIS length or location. This difference likely reflects the greater asymmetry of capacitive and conductance loads between somatodendritic domains and axons in myelinated neurons.

### Mechanisms of somatodendritic impact on AIS plasticity

How does somatodendritic size impact AIS performance? Action potential initiation depends on the speed and magnitude of depolarization in the AIS. Under physiological conditions, depolarization of the AIS starts with passive spread of excitatory synaptic potentials arriving from the soma, and ends with rapid recruitment of local voltage-gated sodium conductances in the AIS. Because voltage attenuation of somatic signals in the thin (∼1.5 µm diameter) AIS is small (∼4% at 85 µm from the soma; see also Kole et al., 2007) and not dependent on neuron size, there is only modest distance-dependent reduction of excitatory drive as the AIS is moved distally from the soma. On the other hand, intrinsic AIS properties that determine the magnitude and rise time of local depolarization from sodium currents are both location and neuron size dependent. Local R_N_, which determines the magnitude of local depolarization, was negatively correlated with AIS distance in small neurons, but increased, and with a progressively steeper relationship, with distance from the soma as neurons increased in size. Similarly, in all but the soma-only model, the effective local τ_M_ in the AIS became faster with distance from the soma, again with progressively steeper dependencies on distance as neurons increased in size. Combined with the reduced impact of somatodendritic capacitance at distal AIS locations (which contributes to the faster effective τ_M_), these intrinsic AIS properties facilitate action potential initiation at distal AIS locations in larger neurons (Fig. 15). Thus, while small neurons gain little, if any, enhancement to excitability from distance-dependent changes in local R_N_ or τ_M_, in larger neurons these AIS properties can more than balance the modest voltage attenuation of somatic depolarizing drive.

Consistent with the impact of neuron size on AIS performance, increasing dendritic capacitance and/or conductance alone mimicked enlarging the dendritic tree. However, changes in dendritic C_M_ were more effective at shifting optimal AIS length and location than were changes of similar magnitude to dendritic R_M_. This likely results because changes in dendritic R_M_, but not C_M_, influence the electrotonic length constant (λ) of dendrites. For instance, in addition to increasing dendritic conductance (shifting optimal AIS locations distally), lowering dendritic R_M_ decreases dendritic λ, thereby reducing the influence of more distal dendritic capacitance on the AIS. Conversely, increasing dendritic R_M_, which shifts optimal AIS locations toward the soma, also increases dendritic λ values and, therefore, enhances the impact of distal dendritic capacitance on AIS performance. Thus, changes in dendritic membrane conductance may to some extent be self-limiting, stabilizing AIS performance in the face of the rapid changes in dendritic membrane conductance typical of neurons *in vivo* (Destexhe et al., 2003). Since dendritic capacitance is expected to be uniform in the absence of structural changes to the dendritic tree, it is likely that plastic changes to AIS architecture are better suited for adapting to structural changes in dendritic morphology, or chronic changes in afferent synaptic drive, than for adapting to changes in membrane conductance.

### The functional role of AIS plasticity

Throughout this article, we have arbitrarily referred to those AIS lengths and locations having minimal rheobase currents as being “optimal,” “favored,” or “preferred.” However, there is no evidence that neurons strive to maximize their intrinsic excitability. On the contrary, neurons use numerous intrinsic and extrinsic mechanisms to effectively dampen excitability. More practically, there is little evidence that AIS architectures *in vivo* maximize excitability. For instance, in the developing visual cortex, the AISs of smaller L2/3 neurons tend to be longer than those of larger L5 neurons (Gutzmann et al., 2014), while both pyramidal neurons and Purkinje neurons have AIS starting locations within a few micrometers of the soma (Khaliq and Raman, 2006; Palmer et al., 2010; Galiano et al., 2012; Harty et al., 2013; Gutzmann et al., 2014; Hamada and Kole, 2015; but see Thome et al., 2014), a structural arrangement that is expected to limit, rather than enhance, the excitability of these relatively large neurons. A more plausible hypothesis is that AIS plasticity, primarily through changes in AIS length, acts as a homeostatic mechanism to fine-tune excitability at relatively slow timescales. This type of AIS plasticity appears to be robust during development (Kaphzan et al., 2011, 2013; Gutzmann et al., 2014; Kuba et al., 2014) but is more modest in adulthood (Gutzmann et al., 2014), with only limited plasticity observed in mature animals following traumatic brain injury (Baalman et al., 2013), pharmacologically induced seizures (Harty et al., 2013), or demyelination (Hamada and Kole, 2015).

Plasticity of AIS location, which itself has only a modest impact on intrinsic excitability, may be more important for tuning frequency response characteristics of neurons. Simulations by Kuba et al. (2006) found that a short AIS, positioned distally from the soma (as found in high-frequency-responsive neurons in the avian nucleus laminaris) enhanced the fidelity of action potential generation in response to simulated high-frequency synaptic input. Since somatodendritic morphology influences AIS performance in the frequency domain (Hay et al., 2013; Eyal et al., 2014), it is possible that neurons adjust AIS location to stabilize frequency response characteristics following changes in somatodendritic morphology. Alternatively, it is possible that AIS translocation could have indirect effects on excitability by changing the spatial relationship between axoaxonic GABAergic inputs and the AIS (Muir and Kittler, 2014; Wefelmeyer et al., 2015).

Overall, our results demonstrate the substantial impact that somatodendritic morphology will have on the direction and magnitude of excitability changes following AIS plasticity. Additional studies that directly test for correlations of AIS architecture, dendritic morphology, and *in vivo* neuronal output, under baseline conditions and following manipulations of afferent input, will be needed to clarify the physiological impact of the different forms of AIS plasticity during normal neuronal development and following injury or disease.

## Synthesis

The decision was a result of the Reviewing Editor Marlene Bartos and the peer reviewers coming together and discussing their recommendations until a consensus was reached. A fact-based synthesis statement explaining their decision and outlining what is needed to prepare a revision is listed below. The following reviewers agreed to reveal their identity: Arnd Roth, Imre Vida

The work was identified as solid study, demonstrating in a detailed compartmental model the dependence of neuronal excitability on the properties of the axon initial segment (AIS) and on the morphological characteristics of the somato-dendritic domain. The work was furthermore judged as highly convincing, the presentation of the data as excellent and the conclusions as well supported by the data. Finally reviewer #1 judged the study as timely because it complements and links recent experimental studies showing that dynamic changes to the length and/or position of the AIS can regulate neuronal excitability. Both reviewers formulated some concerns which are formulated below and should be addressed by the authors.

Reviewer #1:

This is solid work, demonstrating in reduced and detailed compartmental models that the dependence of neuronal excitability on the length and position of the axon initial segment (AIS) can change, even qualitatively, depending on the size of the dendritic tree. The study is timely because it nicely complements and links recent experimental studies showing that dynamic changes to the length and/or position of the AIS can regulate neuronal excitability, and modelling studies showing that the size of the dendritic tree has important consequences for the frequency response of neurons. However, several issues should be addressed.

Major comments

1. Which channel models were used? Hallermann et al. (2012) is mentioned on line 97 in connection with conductance densities, but it is not clear whether the present study also uses their channel models and, if yes, which of them. What are the original sources of the sodium and potassium channel models used? How exactly was their kinetics scaled for the temperature of 37 degC?

2. The authors should test the robustness of their basic results by comparing simulations using different sets of kinetic models of sodium and potassium channels, including classical ones such as the squid axon Hodgkin-Huxley model. (To keep things simple, this test of robustness could be limited to basic results such as those in Figure 2 and in Figure 10.)

3. This test for robustness should also include the case that the ratio of conductance densities in the AIS to those in the soma is smaller than in the present model (e.g. Schmidt-Hieber &amp; Bischofberger, 2010; Fleidervish et al., 2010).

4. The steady-state voltage attenuation from the soma to the AIS (Fig. 11A) is small, but this was measured far from AP threshold. How does the steady-state voltage attenuation from the soma to the AIS look like when it is measured at a more relevant membrane potential for AP initiation, e.g. closer to, but clearly below, AP threshold? Close to threshold, potassium channel activation and inactivation might influence the voltage attenuation in a voltage- dependent manner. This might be a key mechanism influencing the dependence of excitability on AIS length and location. Perhaps related to this, why is there an (albeit weak) dependence of the soma-AIS voltage attenuation on the number of dendrites (Figure 11A)?

5. Is the steady-state voltage attenuation from the soma to the axon consistent with direct experimental measurements of the space constant of voltage attenuation in axons?

6. Line 417ff: Changing Rm also changes the membrane time constant, which (given the stimulus current pulse duration used by the authors) is likely to be one of the reasons why the timing of the AP varies across conditions in Figure 9B. Changing the timing of the AP at threshold will differentially engage the kinetics of activation (and inactivation, where appropriate) of the sodium and potassium channels generating the AP. This may be one of the mechanisms for the Rm dependence observed (Figure 9), and it might be strongly dependent on the particular choice of the kinetic models of sodium and potassium channels (see points 1-3 above).

Minor points

Line 158: Figure 2B,C should be Figure 2A,B. How exactly were the rheobase currents normalized?

Line 173ff, Figure 3A: Occasionally the APs fail to actively propagate to the soma. This should be mentioned and discussed. (It is unusual in many cell types but does occasionally occur, for example, in turtle pyramidal cells.)

Reviewer #2:

This study addresses how morphological and functional properties of the axon initial segment (AIS), in the context of activity dependent plasticity, determine action potential initiation. Using abstract single cell models, the authors first systematically analyze how parameters of the AIS in combination with changes in the somato-dendritic domain determine the current threshold (rheobase). Subsequently, they use morphologically-based detailed multi-compartment models to confirm the relevance of their results for major principal cell types.

The main finding of the study is that length and location of the AIS differentially influence the rheobase depending on the size and structure of the somato-dendritic domain of the neurons: while in small neurons a relatively short and proximal AIS is optimal for the activation of the neurons, unexpectedly in large neurons a longer and more distally located AIS is advantages.

The results are convincing, the presentation of the data is excellent and the conclusions are well supported by the data set. While some aspects may appear at first counter-intuitive, results and conclusions of the study will provide an important reference for the interpretation of the morphological differences as well as the plastic changes of the AIS observed in various neuron types of the brain.

I have only a few minor concerns and comments to the manuscript:

1) The distally located AIS is described as "optimal" for larger neurons, as it uncouples the AP initiation site from the large somato-dendritic capacitive load enabling a lower threshold. However under these conditions APs apparently cannot backpropagate to the soma (see e.g. Fig 3A, 4 Dendrites, AIS at 70 um). The strong attenuation of the somatic AP suggests that the uncoupling of the somato-dendritic domain and the AIS is physiologically not realistic. Please analyze and discuss this aspect in more detail.

2) The expression "capacitive shielding" used of the electrotonic uncoupling of the AIs from the somato-dendritic capacitive "load" is misleading as it implies some protective mechanisms by a certain capacitance. Please reword.

3) Please illustrate action potentials generated in the AIS and the soma of the morphologically-based single cell models.

4) What is the basis for the increasing Rin for distal locations of the AIS in small neurons? Please try to give an intuitive explanation.

5) The axon typically emerges from the soma or a dendrite with a short, strongly tapering segment, the axon hillock. Why was this special morphological feature not considered in the models? How would the presence of the hillock influence the results?

## References

[B1] Adachi R, Yamada R, Kuba H (2015) Plasticity of the axonal trigger zone. Neuroscientist 21:255–265. 10.1177/1073858414535986 24847046

[B2] Baalman KL, Cotton RJ, Rasband SN, Rasband MN (2013) Blast wave exposure impairs memory and decreases axon initial segment length. J Neurotrauma 30:741-751. 10.1089/neu.2012.2478 23025758 PMC3941920

[B3] Baranauskas G, David Y, Fleidervish IA (2013) Spatial mismatch between the Na^+^ flux and spike initiation in axon initial segment. Proc Natl Acad Sci U S A 110:4051-4056. 10.1073/pnas.1215125110 23341597 PMC3593864

[B4] Bi GQ, Poo MM (1998) Synaptic modifications in cultured hippocampal neurons: dependence on spike timing, synaptic strength, and postsynaptic cell type. J Neurosci 18:10464-10472. 9852584 10.1523/JNEUROSCI.18-24-10464.1998 PMC6793365

[B5] Carnevale NT, Hines ML (2006) The neuron book. New York: Cambridge University Press 10.1017/cbo9780511541612

[B6] Chand AN, Galliano E, Chesters RA, Grubb MS (2015) A distinct subtype of dopaminergic interneuron displays inverted structural plasticity at the axon initial segment. J Neurosci 35:1573-1590. 10.1523/JNEUROSCI.3515-14.2015 25632134 PMC4308603

[B7] Destexhe A, Rudolph M, Paré D (2003) The high-conductance state of neocortical neurons in vivo. Nat Rev Neurosci 4:739-751. 10.1038/nrn1198 12951566

[B8] Evans MD, Sammons RP, Lebron S, Dumitrescu AS, Watkins TB, Uebele VN, Renger JJ, Grubb MS (2013) Calcineurin signaling mediates activity-dependent relocation of the axon initial segment. J Neurosci 33:6950-6963. 10.1523/JNEUROSCI.0277-13.2013 23595753 PMC3743026

[B9] Eyal G, Mansvelder HD, de Kock CP, Segev I (2014) Dendrites impact the encoding capabilities of the axon. J Neurosci 34:8063-8071. 10.1523/JNEUROSCI.5431-13.2014 24920612 PMC6608238

[B10] Galiano MR, Jha S, Ho TS, Zhang C, Ogawa Y, Chang KJ, Stankewich MC, Mohler PJ, Rasband MN (2012) A distal axonal cytoskeleton forms an intra-axonal boundary that controls axon initial segment assembly. Cell 149:1125-1139. 10.1016/j.cell.2012.03.039 22632975 PMC3361702

[B11] Grubb MS, Burrone J (2010) Activity-dependent relocation of the axon initial segment fine-tunes neuronal excitability. Nature 465:1070-1074. 10.1038/nature09160 20543823 PMC3196626

[B12] Grubb MS, Shu Y, Kuba H, Rasband MN, Wimmer VC, Bender KJ (2011) Short- and long-term plasticity at the axon initial segment. J Neurosci 31:16049-16055. 10.1523/JNEUROSCI.4064-11.2011 22072655 PMC3232445

[B13] Gutzmann A, Ergül N, Grossmann R, Schultz C, Wahle P, Engelhardt M (2014) A period of structural plasticity at the axon initial segment in developing visual cortex. Front Neuroanat 8:11. 10.3389/fnana.2014.00011 24653680 PMC3949221

[B14] Hallermann S, de Kock CP, Stuart GJ, Kole MH (2012) State and location dependence of action potential metabolic cost in cortical pyramidal neurons. Nat Neurosci 15:1007-1014. 10.1038/nn.3132 22660478

[B15] Hamada MS, Kole MH (2015) Myelin loss and axonal ion channel adaptations associated with gray matter neuronal hyperexcitability. J Neurosci 35:7272-7286. 10.1523/JNEUROSCI.4747-14.2015 25948275 PMC4420788

[B16] Harty RC, Kim TH, Thomas EA, Cardamone L, Jones NC, Petrou S, Wimmer VC (2013) Axon initial segment structural plasticity in animal models of genetic and acquired epilepsy. Epilepsy Res 105:272-279. 10.1016/j.eplepsyres.2013.03.004 23602553

[B17] Hay E, Schürmann F, Markram H, Segev I (2013) Preserving axosomatic spiking features despite diverse dendritic morphology. J Neurophysiol 109:2972-2981. 10.1152/jn.00048.2013 23536715

[B18] Hemond P, Migliore M, Ascoli GA, Jaffe DB (2009) The membrane response of hippocampal CA3b pyramidal neurons near rest: heterogeneity of passive properties and the contribution of hyperpolarization-activated currents. Neuroscience 160:359-370. 10.1016/j.neuroscience.2009.01.082 19232379 PMC3560914

[B19] Holmes WR (1989) The role of dendritic diameters in maximizing the effectiveness of synaptic inputs. Brain Res 478:127-137. 10.1016/0006-8993(89)91484-4 2538199

[B20] Kaphzan H, Buffington SA, Jung JI, Rasband MN, Klann E (2011) Alterations in intrinsic membrane properties and the axon initial segment in a mouse model of Angelman syndrome. J Neurosci 31:17637-17648. 10.1523/JNEUROSCI.4162-11.2011 22131424 PMC3483031

[B21] Kaphzan H, Buffington SA, Ramaraj AB, Lingrel JB, Rasband MN, Santini E, Klann E (2013) Genetic reduction of the α1 subunit of Na/K-ATPase corrects multiple hippocampal phenotypes in Angelman syndrome. Cell Rep 4:405-412. 10.1016/j.celrep.2013.07.005 23911285 PMC3756897

[B22] Khaliq ZM, Raman IM (2006) Relative contributions of axonal and somatic Na channels to action potential initiation in cerebellar Purkinje neurons. J Neurosci 26:1935-1944. 10.1523/JNEUROSCI.4664-05.2006 16481425 PMC6674931

[B23] Kole MH, Stuart GJ (2012) Signal processing in the axon initial segment. Neuron 73:235-247. 10.1016/j.neuron.2012.01.007 22284179

[B24] Kole MH, Hallermann S, Stuart GJ (2006) Single I_h_ channels in pyramidal neuron dendrites: properties, distribution, and impact on action potential output. J Neurosci 26:1677-1687. 10.1523/JNEUROSCI.3664-05.2006 16467515 PMC6793638

[B25] Kole MH, Letzkus JJ, Stuart GJ (2007) Axon initial segment Kv1 channels control axonal action potential waveform and synaptic efficacy. Neuron 55:633-647. 10.1016/j.neuron.2007.07.031 17698015

[B26] Kole MH, Ilschner SU, Kampa BM, Williams SR, Ruben PC, Stuart GJ (2008) Action potential generation requires a high sodium channel density in the axon initial segment. Nat Neurosci 11:178-186. 10.1038/nn2040 18204443

[B27] Kuba H (2012) Structural tuning and plasticity of the axon initial segment in auditory neurons. J Physiol 590:5571-5579. 10.1113/jphysiol.2012.237305 23027822 PMC3528978

[B28] Kuba H, Ishii TM, Ohmori H (2006) Axonal site of spike initiation enhances auditory coincidence detection. Nature 444:1069-1072. 10.1038/nature05347 17136099

[B29] Kuba H, Oichi Y, Ohmori H (2010) Presynaptic activity regulates Na(+) channel distribution at the axon initial segment. Nature 465:1075-1078. 10.1038/nature09087 20543825

[B30] Kuba H, Adachi R, Ohmori H (2014) Activity-dependent and activity-independent development of the axon initial segment. J Neurosci 34:3443-3453. 10.1523/JNEUROSCI.4357-13.2014 24573300 PMC6795309

[B31] Larkum ME, Zhu JJ, Sakmann B (1999) A new cellular mechanism for coupling inputs arriving at different cortical layers. Nature 398:338-341. 10.1038/18686 10192334

[B32] Mainen ZF, Sejnowski TJ (1996) Influence of dendritic structure on firing pattern in model neocortical neurons. Nature 382:363-366. 10.1038/382363a0 8684467

[B33] Martone ME, Gupta A, Wong M, Qian X, Sosinsky G, Ludäscher B, Ellisman MH (2002) A cell-centered database for electron tomographic data. J Struct Biol 138:145-155. 10.1016/s1047-8477(02)00006-0 12160711

[B34] Muir J, Kittler JT (2014) Plasticity of GABAA receptor diffusion dynamics at the axon initial segment. Front Cell Neurosci 8:151. 10.3389/fncel.2014.00151 24959118 PMC4051194

[B35] Palmer LM, Stuart GJ (2006) Site of action potential initiation in layer 5 pyramidal neurons. J Neurosci 26:1854-1863. 10.1523/JNEUROSCI.4812-05.2006 16467534 PMC6793621

[B36] Palmer LM, Clark BA, Gründemann J, Roth A, Stuart GJ, Häusser M (2010) Initiation of simple and complex spikes in cerebellar Purkinje cells. J Physiol 588:1709-1717. 10.1113/jphysiol.2010.188300 20351049 PMC2887989

[B37] Roth A, Häusser M (2001) Compartmental models of rat cerebellar Purkinje cells based on simultaneous somatic and dendritic patch-clamp recordings. J Physiol 535:445-472. 10.1111/j.1469-7793.2001.00445.x 11533136 PMC2278793

[B38] Schmidt-Hieber C, Bischofberger J (2010) Fast sodium channel gating supports localized and efficient axonal action potential initiation. J Neurosci 30:10233-10242. 10.1523/JNEUROSCI.6335-09.2010 20668206 PMC6633381

[B39] Schmidt-Hieber C, Jonas P, Bischofberger J (2007) Subthreshold dendritic signal processing and coincidence detection in dentate gyrus granule cells. J Neurosci 27:8430-8441. 10.1523/JNEUROSCI.1787-07.2007 17670990 PMC6673070

[B40] Stuart G, Spruston N (1998) Determinants of voltage attenuation in neocortical pyramidal neuron dendrites. J Neurosci 18:3501-3510. 9570781 10.1523/JNEUROSCI.18-10-03501.1998 PMC6793161

[B41] Thome C, Kelly T, Yanez A, Schultz C, Engelhardt M, Cambridge SB, Both M, Draguhn A, Beck H, Egorov AV (2014) Axon-carrying dendrites convey privileged synaptic input in hippocampal neurons. Neuron 83:1418-1430. 10.1016/j.neuron.2014.08.013 25199704

[B42] van Elburg RA, van Ooyen A (2010) Impact of dendritic size and dendritic topology on burst firing in pyramidal cells. PLoS Comput Biol 6:e1000781. 10.1371/journal.pcbi.1000781 20485556 PMC2869305

[B43] van Ooyen A, Duijnhouwer J, Remme MW, van Pelt J (2002) The effect of dendritic topology on firing patterns in model neurons. Network: Computation in Neural Systems 13:311-325. 10.1088/0954-898x/13/3/304 12222816

[B44] Wefelmeyer W, Cattaert D, Burrone J (2015) Activity-dependent mismatch between axo-axonic synapses and the axon initial segment controls neuronal output. Proc Natl Acad Sci U S A 112:9757-9762. 10.1073/pnas.1502902112 26195803 PMC4534224

